# SSB deficiency-induced R-loop accumulation triggers podocyte inflammation in DKD

**DOI:** 10.3389/fimmu.2026.1911700

**Published:** 2026-08-20

**Authors:** Jixin Xing, Shanbo Wang

**Affiliations:** Department of Nephrology, the 960th Hospital of the People’s Liberation Army, Jinan, China

**Keywords:** cGAS-dependent inflammatory signaling, diabetic kidney disease, machine learning, podocyte inflammation, R-loop, single-cell transcriptomics, SSB

## Abstract

**Introduction:**

Diabetic kidney disease (DKD) is fundamentally a podocytopathy in which sterile inflammation plays a central pathogenic role, yet the upstream triggers that initiate inflammatory cascades in podocytes remain elusive. R-loops are critical regulators of genomic stability, and their pathological accumulation triggers DNA damage and innate immune activation. Whether R-loop dysregulation contributes to podocyte-driven inflammation in DKD is unknown.

**Methods:**

We integrated single-cell transcriptomic profiling, dual machine learning algorithms, and functional experiments to dissect the R-loop regulatory network in the diabetic kidney.

**Results:**

Integrated analysis of human diabetic kidney single-cell RNA-seq data revealed a globally compromised R-loop regulatory network selectively within podocytes. Intersection of podocyte-specific transcriptomic shifts with validated R-loop regulators identified 93 candidate genes, from which dual machine learning algorithms pinpointed SSB (Sjögren syndrome antigen B) as the principal podocyte-selective R-loop resolver and a superior diagnostic biomarker (AUC = 0.983). SSB expression was selectively downregulated in diabetic podocytes and showed the strongest positive correlation with the R-loop resolution module. Mechanistically, SSB loss impaired RNA splicing and stability pathways, leading to aberrant R-loop accumulation that activated the cGAS-dependent inflammatory signaling in podocytes. In two murine DKD models and high glucose-challenged podocytes, SSB was markedly reduced. Remarkably, SSB knockdown in podocytes alone sufficed to trigger R-loop accumulation and pro-inflammatory cytokine expression, whereas both RNase H1-mediated R-loop removal and cGAS co-depletion blunted this response.

**Discussion:**

These findings suggest that an SSB-governed R-loop -cGAS -inflammatory signaling axis may link genomic instability to podocyte inflammation and contribute to DKD progression, nominating R-loop homeostasis as a previously unrecognized potential therapeutic target.

## Introduction

Diabetic kidney disease (DKD) is the leading cause of end-stage renal disease worldwide and is increasingly recognized as a chronic inflammatory disorder rather than a purely metabolic or hemodynamic condition (1–3). Podocytes, the terminally differentiated visceral epithelial cells of the glomerulus, are central to the pathogenesis of DKD, which is often described as a “podocytopathy” (4, 5). These cells possess a highly restricted regenerative capacity, rendering them critically dependent on genomic integrity for long-term survival. Consequently, podocyte injury and depletion are primary drivers of proteinuria and progressive glomerulosclerosis. Over the past decade, compelling evidence has demonstrated that podocyte damage triggers local innate immune responses, including the release of pro-inflammatory cytokines, which amplify tissue injury and fibrosis (3, 6, 7). However, the molecular events that couple initial podocyte stress to the activation of inflammatory programs remain incompletely understood.

R-loops are dynamic three-stranded nucleic acid structures composed of an RNA: DNA hybrid and a displaced single-stranded DNA strand (8). They form co-transcriptionally and participate in physiological processes such as transcription regulation and immunoglobulin class switching (9). Nevertheless, when R-loop formation, processing, and resolution are imbalanced, these structures accumulate pathologically and become potent sources of genomic instability (10). Persistent R-loops induce replication-transcription conflicts, DNA double-strand breaks, and cellular senescence. Emerging studies have revealed that unresolved R-loops and their associated DNA damage can lead to the leakage of self-nucleic acids into the cytosol, where they act as danger-associated molecular patterns (DAMPs) that activate the cGAS-dependent innate immune pathway (11, 12). This nucleic acid-sensing mechanism establishes a direct mechanistic link between nuclear R-loop dysregulation and cytosolic inflammatory signaling. Given the enormous transcriptional demand imposed on podocytes to maintain the glomerular filtration barrier and the critical importance of genomic stability for their survival, we hypothesized that disruption of R-loop homeostasis may be a fundamental pathogenic mechanism that triggers podocyte injury and fuels the chronic inflammatory milieu of DKD.

To address this hypothesis, we first mapped the transcriptomic landscape of the human diabetic kidney microenvironment using single-cell RNA sequencing (scRNA-seq) data. We systematically evaluated the overall R-loop regulatory capacity of podocytes across diabetic and control samples and identified a profound collapse of the R-loop regulatory network specifically in diabetic podocytes. By integrating podocyte-specific differentially expressed genes with a comprehensive repertoire of validated R-loop regulators from the R-loopBase database, we distilled a refined candidate set that encapsulates the core dysregulated machinery. Leveraging dual machine learning algorithms on an independent diabetic glomerular transcriptomic cohort, we pinpointed SSB (Sjögren syndrome antigen B, also known as La protein) as the most robust podocyte-selective R-loop resolver and a highly sensitive diagnostic biomarker. Our computational analyses indicated that SSB deficiency impairs RNA splicing and stability pathways in podocytes, leading to R-loop accumulation and subsequent activation of the cGAS-dependent inflammatory signaling axis. We observed SSB downregulation in two murine DKD model, and further demonstrated in cultured podocytes that high glucose suppresses SSB expression and that SSB knockdown is sufficient to induce R-loop accumulation and cGAS-dependent inflammation, while RNase H1-mediated R-loop removal or Cgas depletion reverses this phenotype, indicating a cell-autonomous requirement for SSB. Collectively, this study suggests an SSB–R-loop–cGAS-dependent inflammatory signaling axis that may translate genomic instability into podocyte inflammation, highlighting R-loop homeostasis as a potential therapeutic vulnerability in DKD.

## Results

### Single-cell transcriptomic landscape reveals a compromised R-loop regulatory network in diabetic podocytes

**Figure 1:**
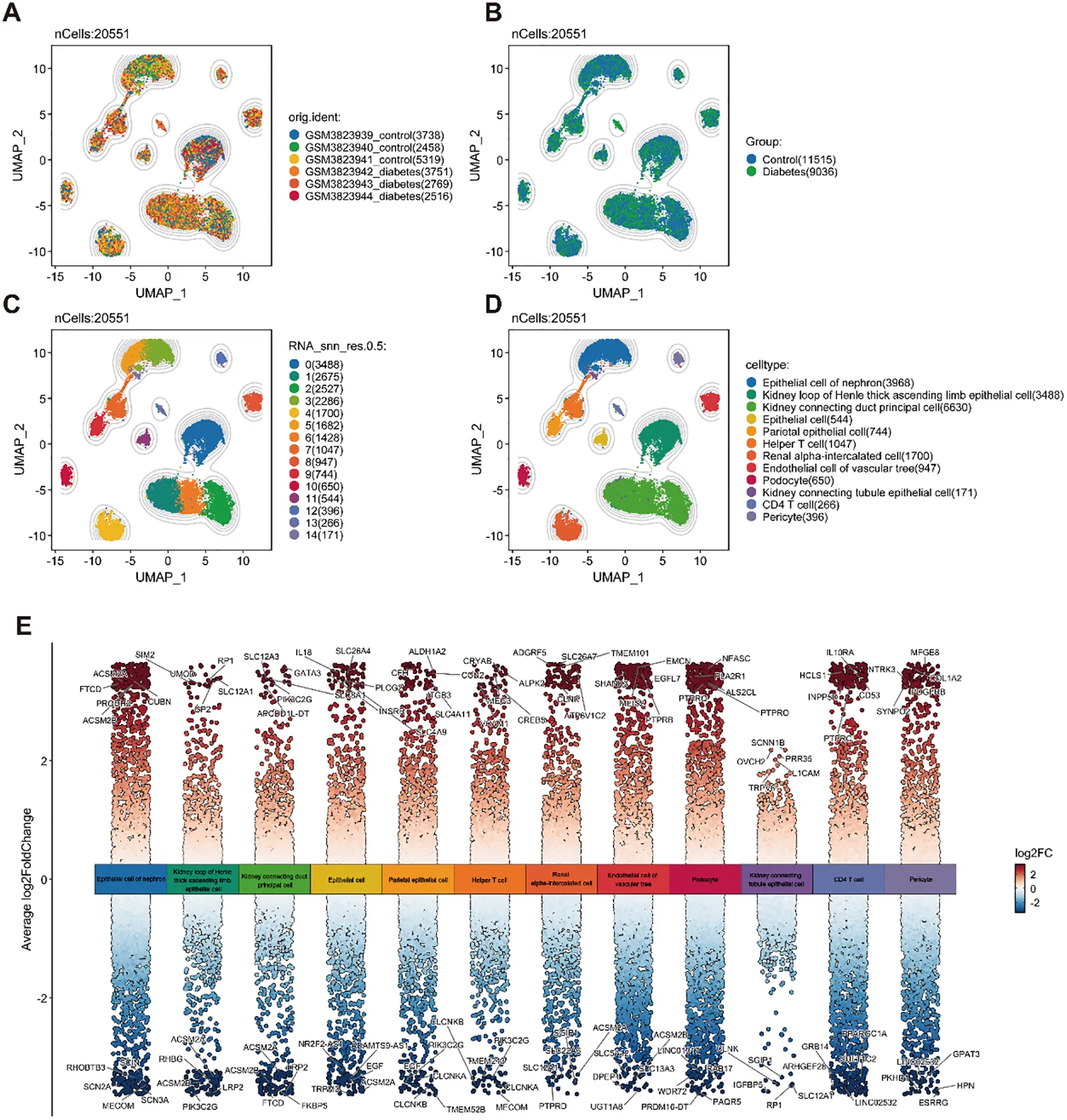
Single-cell transcriptomic landscape and cell-type annotation of the diabetic kidney microenvironment. **(A, B)** UMAP embeddings demonstrating the robust batch-effect correction and integration by Harmony, color-coded by the six biological sample origins (orig.ident) **(A)** and clinical groups (Control vs. DKD) **(B)**. **(C)** Uniform Manifold Approximation and Projection (UMAP) visualization of 15 initial cell clusters identified at a resolution of 0.5. **(D)** UMAP plot displaying the 12 major renal cell lineages annotated via the ACT method and canonical marker matching. **(E)** Volcano plot highlighting the top upregulated and downregulated differentially expressed genes (DEGs) across the 12 distinct cell subpopulations.

Mapping the complex transcriptomic landscape of the diabetic kidney microenvironment was initially achieved by analyzing the single-cell RNA sequencing (scRNA-seq) dataset GSE131882 from the GEO database (13). Following rigorous quality control, the Harmony algorithm was applied to integrate the data across six distinct biological samples (*orig.ident*) and two clinical groups (Control and Diabetes). UMAP projections demonstrated a highly effective integration, successfully eliminating batch effects while preserving intrinsic biological variance (**Figures 1A, B**). Using the clustree algorithm, an optimal resolution of 0.5 was determined, partitioning the cells into 15 initial clusters (clusters 0-14) (**Figure 1C**). Subsequently, differential gene expression for each cluster was computed via the COSG algorithm. Coupling this with the ACT automated annotation tool and canonical marker matching, these 15 clusters were precisely consolidated into 12 distinct renal cell lineages. These predominantly encompassed Epithelial cell of nephron, Kidney loop of Henle thick ascending limb epithelial cell, Kidney connecting duct principal cell, Epithelial cell, Parietal epithelial cell, Helper T cell, Renal alpha-intercalated cell, Endothelial cell of vascular tree, Podocyte, Kidney connecting tubule epithelial cell, CD4 T cell, Pericyte (**Figure 1D**). Podocyte identity was validated by expression of canonical markers: NPHS1, NPHS2, WT1 and SYNPO (**Supplementary Figure 1**). Widespread transcriptomic reprogramming across these diverse lineages was captured via volcano plots, highlighting the top differentially expressed genes (DEGs) driven by the diabetic milieu (**Figure 1E**).

**Figure 2:**
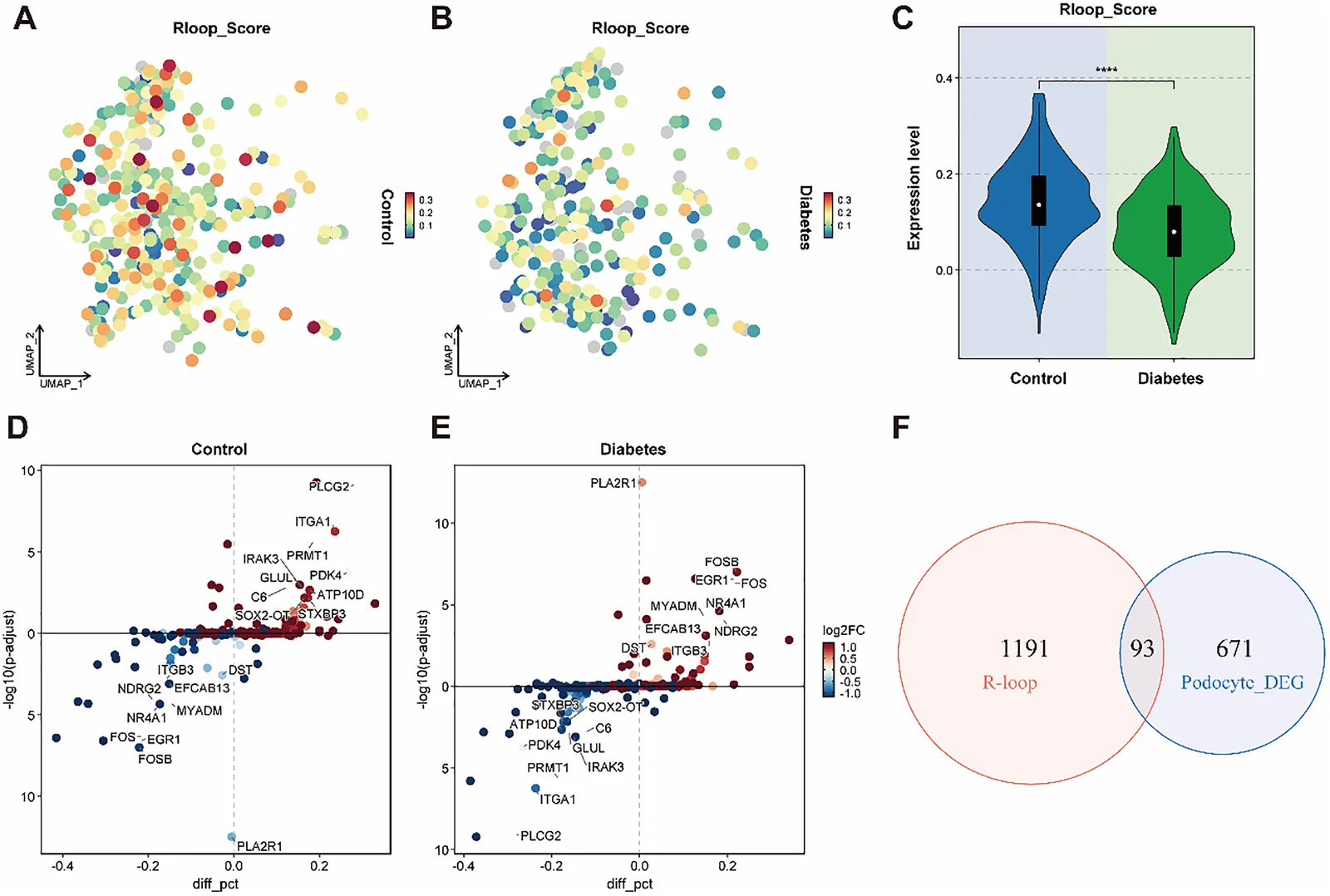
Disruption of R-loop homeostasis and identification of dysregulated R-loop regulators in diabetic podocytes. **(A, B)** Feature plots visualizing the overall R-loop regulatory score mapped onto the podocyte subpopulation in the Control **(A)** and DKD **(B)** groups. **(C)** Violin plot quantifying the significant reduction of the overall R-loop regulatory score in diabetic podocytes compared to non-diabetic controls. **(D, E)** Volcano plots illustrating the transcriptomic shifts in podocytes, depicting significant DEGs upregulated in the Control group **(D)** and DKD group **(E)**. **(F)** Venn diagram demonstrating the intersection between 764 podocyte-specific DEGs and 1,284 curated R-loop regulatory genes, yielding 93 primary candidate genes. ****P < 0.0001.

Diabetic kidney disease (DKD) is now recognized as a chronic inflammatory disease, in which podocyte injury-driven inflammatory responses play a key role in disease progression. DKD is fundamentally a “podocytopathy” (14), wherein the injury and loss of these terminally differentiated visceral epithelial cells directly dictate the disease course. Given their limited regenerative capacity, podocytes are acutely reliant on genomic stability for survival (15). R-loops—dynamic three-stranded nucleic acid structures consisting of an RNA: DNA hybrid and a displaced single-stranded DNA—are essential for normal transcription (9). However, their pathological dysregulation acts as a potent instigator of DNA damage and cellular senescence (10), two processes that serve as critical upstream events in the activation of chronic inflammatory cascades. We therefore hypothesize that disruption of R-loop homeostasis may constitute a central pathogenic mechanism that triggers podocyte injury and drives the chronic inflammatory progression of DKD. Extracting specifically the podocyte subpopulation, we evaluated their overall R-loop regulatory capacity using the AddModuleScore function in Seurat, based on a comprehensive set of 1,284 validated R-loop regulatory genes acquired from the R-loopBase database (16). Intriguingly, UMAP visualization mapping these scores exhibited a stark contrast between the clinical groups (**Figures 2A, B**). Quantitative evaluation via violin plots further corroborated that the overall R-loop regulatory score was markedly diminished in diabetic podocytes compared to their non-diabetic counterparts (**Figure 2C**). This significant reduction strongly implies a functional collapse of the intrinsic R-loop processing and resolving machinery under high-glucose stress, rendering the cells highly susceptible to subsequent R-loop accumulation and genomic injury.

Driven by this striking collapse of the R-loop regulatory network, we sought to isolate the specific molecular culprits responsible for this podocyte impairment. Targeted differential expression analysis within the podocyte cluster revealed robust transcriptional shifts, yielding a total of 764 podocyte-specific DEGs between the Control and Diabetes groups (**Figures 2D, E**). By intersecting these 764 DEGs with the established 1,284 R-loop regulatory genes, we distilled a highly refined panel of 93 critical intersection genes (**Figure 2F**). This specific gene set encapsulates the core components of the dysregulated R-loop machinery and provides a robust foundational candidate pool for the downstream identification of key mechanistic targets n DKD-induced podocyte inflammation.

### Dual machine learning algorithms pinpoint podocyte-specific SSB as a core R-loop resolver in DKD

**Figure 3:**
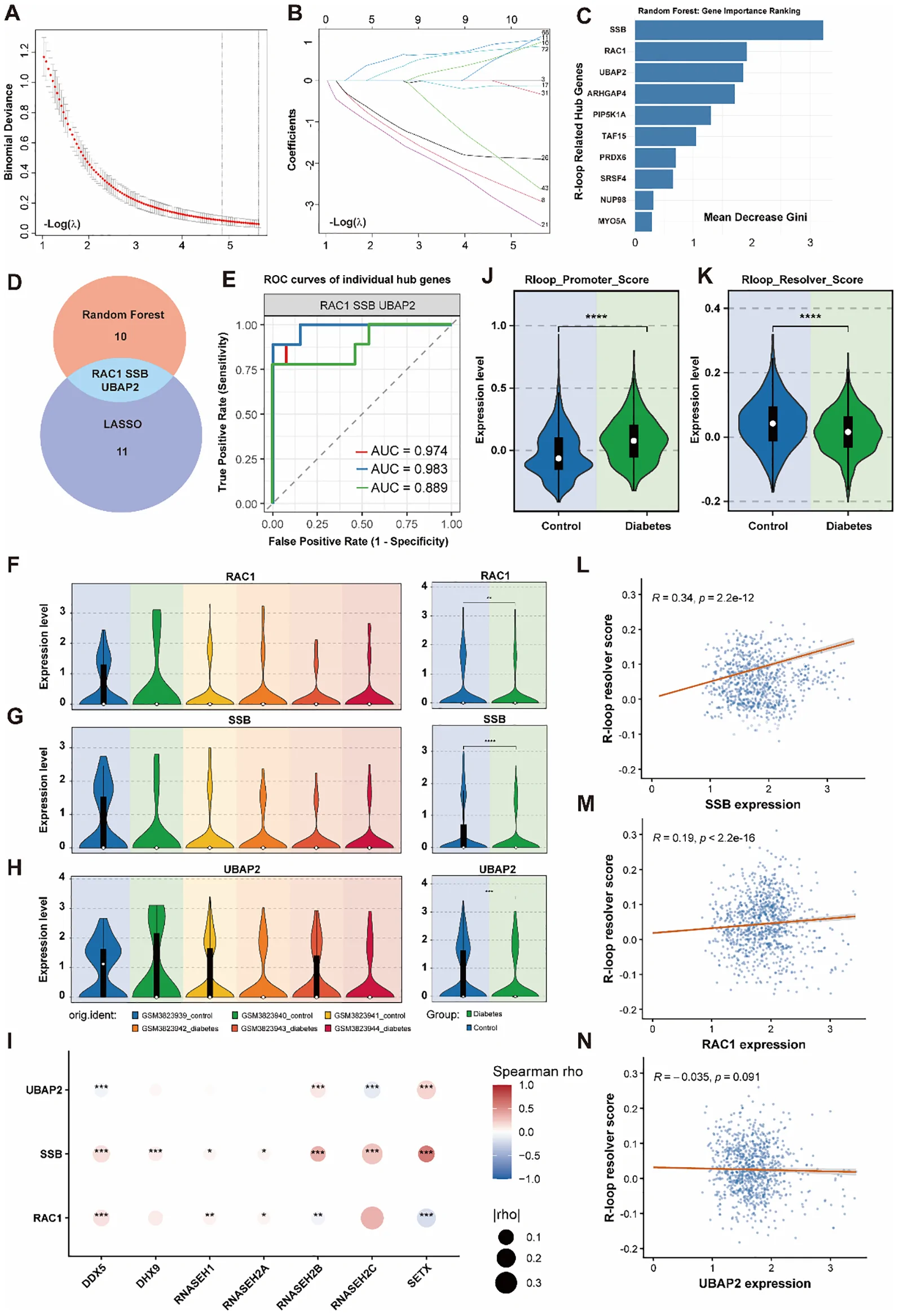
Machine learning-based identification of SSB as a core diagnostic biomarker dictating R-loop resolution. **(A, B)** Feature selection via LASSO regression model using the GSE30122 bulk RNA-seq dataset. The plots display the binomial deviance **(A)** and coefficient profiles **(B)** plotted against Log(λ), filtering 13 essential features. **(C)** Random Forest (RF) algorithm ranking the importance of variables based on the Mean Decrease Gini index, identifying the top 10 candidate genes.**(D)** Venn diagram illustrating the intersection of LASSO and RF outputs, pinpointing three central hub genes: RAC1, SSB, and UBAP2. **(E)** Receiver Operating Characteristic (ROC) curves evaluating the diagnostic efficacy of the three hub genes, with SSB exhibiting the highest AUC (0.983). **(F-H)** Violin plots comparing the expression levels of the three hub genes across individual samples and clinical groups at the single-cell resolution.**(I)** Dot plot showing the significant co-expression correlation between SSB and well-established genes responsible for pathological R-loop accumulation. **(J, K)** Violin plots evaluating the decoupled R-loop functional modules in podocytes. The Rloop_Promoter score is significantly elevated **(J)**, while the Rloop_Resolver score is markedly diminished in the DKD group **(K)**. **(L-N)** Correlation analysis between the three hub genes and the Rloop_Resolver score, revealing that SSB exhibits the most profound positive correlation, functionally classifying it as a core resolver. *P < 0.05; **P < 0.01; ***P < 0.001; and ****P < 0.0001.

Transitioning from the initial single-cell transcriptomic candidate pool, we aimed to identify the most robust clinical biomarkers among the 93 intersecting genes. Utilizing the bulk RNA-seq dataset GSE30122 (17) as a training cohort, dual machine learning algorithms were deployed for rigorous feature selection. First, the Least Absolute Shrinkage and Selection Operator (LASSO) regression was executed to compress highly collinear variables. Using 10-fold cross-validation, LASSO regression identified 11 genes with non-zero coefficients at lambda.min (detailed cross-validation results in **Supplementary Table 1**). By tuning the penalty parameter (λ), the model minimized the binomial deviance and extracted 13 essential features (**Figures 3A, B**). In parallel, a Random Forest (RF) classifier was constructed to rank the genes based on their predictive importance, subsequently identifying 10 top-tier candidate genes (**Figure 3C**). Intersection of the outputs from both the LASSO and RF models yielded three ultimate hub genes: RAC1, SSB, and UBAP2 (**Figure 3D**). Receiver Operating Characteristic (ROC) curve analysis was then performed to evaluate the diagnostic efficacy of these hub genes. Strikingly, all three genes demonstrated exceptional predictive value, with *SSB* exhibiting the highest Area Under the Curve (AUC) of 0.983 (95% CI: (0.9316-1), p < 0.001), closely followed by RAC1 (AUC = 0.974, 95% CI: (0.9231-1), p < 0.001) and UBAP2 (AUC = 0.889, 95% CI: (0.7264-1), p < 0.001) (**Figure 3E**).

Returning to the single-cell resolution to validate these findings, violin plots across six individual samples and clinical groups revealed that among the three hub genes, *SSB* exhibited the most profound and statistically significant differential expression between diabetic and control podocytes (**Figures 3F-H**). Furthermore, correlation analysis via dot plots demonstrated that *SSB* expression was significantly associated with a panel of known core genes responsible for pathological R-loop accumulation (**Figure 3I**). This finding strongly implicates SSB as a primary molecular hub orchestrating R-loop dynamics in the diabetic microenvironment.

To validate the diagnostic robustness of SSB, we analyzed an independent cohort from GEO (GSE30528). This validation dataset included 13 control and 9 DKD glomerular samples (laser capture microdissection). In this external cohort, SSB maintained robust diagnostic performance with AUC of 0.956 (95% CI: (0.8851-1)), confirming that the diagnostic value of SSB is not a result of overfitting in the training dataset.

Maintenance of R-loop homeostasis dictates that the rate of R-loop formation must be tightly balanced by its timely clearance (9). To dissect the specific topological mechanism driving R-loop accumulation in DKD, we revisited the initial pool of 1,284 R-loop regulators. Rather than classifying the entire dataset, we specifically extracted a curated subset of genes whose biological functions have been explicitly characterized in previous studies. Based on established literature, these functionally validated regulators were then stratified into two opposing modules: “Rloop_Promoters” (factors facilitating hybrid formation) and “Rloop_Resolvers” (factors mediating hybrid clearance). Subsequent scoring of these curated functional modules within the podocyte cluster revealed a pathogenic imbalance under diabetic conditions. Specifically, the *Rloop_Promoter* score was significantly elevated in the Diabetes group (**Figure 3J**), whereas the *Rloop_Resolver* score was markedly diminished (**Figure 3K**). This dual-directional disruption provides compelling computational evidence that the pathological accumulation of R-loops in diabetic podocytes is inherently driven by a compromised resolution capacity.

Driven by this mechanistic insight, we further evaluated the correlation between our three hub genes and the *Rloop_Resolver* module. Crucially, *SSB* displayed the strongest positive correlation with the *Rloop_Resolver* score compared to RAC1 and UBAP2 (**Figures 3L-N**). This profound association structurally classifies SSB as a critical component of the R-loop clearance machinery.

**Figure 4:**
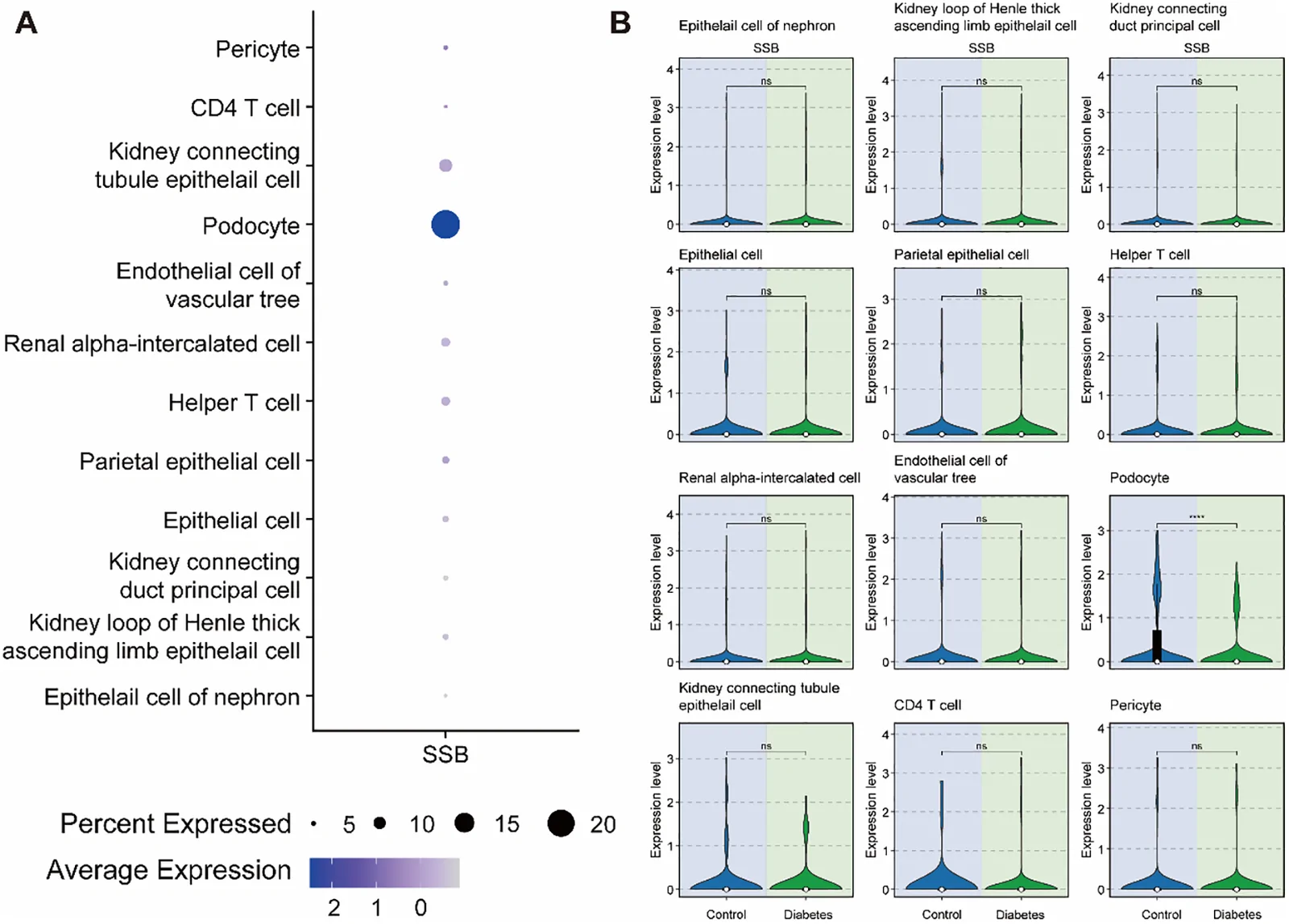
Podocyte-specific transcriptomic downregulation of SSB in diabetic kidney disease. **(A)** Dot plot mapping the expression profile of SSB across all 12 identified renal cell lineages, demonstrating its intrinsically high expression in podocytes. **(B)** Multi-violin plots comparing SSB expression between Control and DKD groups across various cell types. The significant downregulation of SSB is strictly confined to the podocyte cluster, highlighting its cell-type exclusivity in the disease pathogenesis. ****P < 0.0001.

Further investigation into the spatial expression profile of *SSB* underscored its unique biological relevance in the glomerular microenvironment. Dot plot mapping across all identified cell lineages demonstrated that *SSB* is intrinsically highly expressed in podocytes compared to other surrounding cell types (**Figure 4A**). More importantly, multi-violin plot analysis revealed a striking cell-type exclusivity: the significant downregulation of *SSB* observed between the clinical groups was strictly confined to the podocyte cluster, remaining unaltered in other renal lineages (**Figure 4B**). Collectively, these sequential analyses not only prioritize SSB as a highly specific diagnostic biomarker but also establish it as an indispensable “resolver”, the depletion of which leads to R-loop accumulation in diabetic podocytes.

### SSB-mediated disruption of RNA metabolism links R-loop accumulation to cGAS-dependent inflammatory signaling

**Figure 5:**
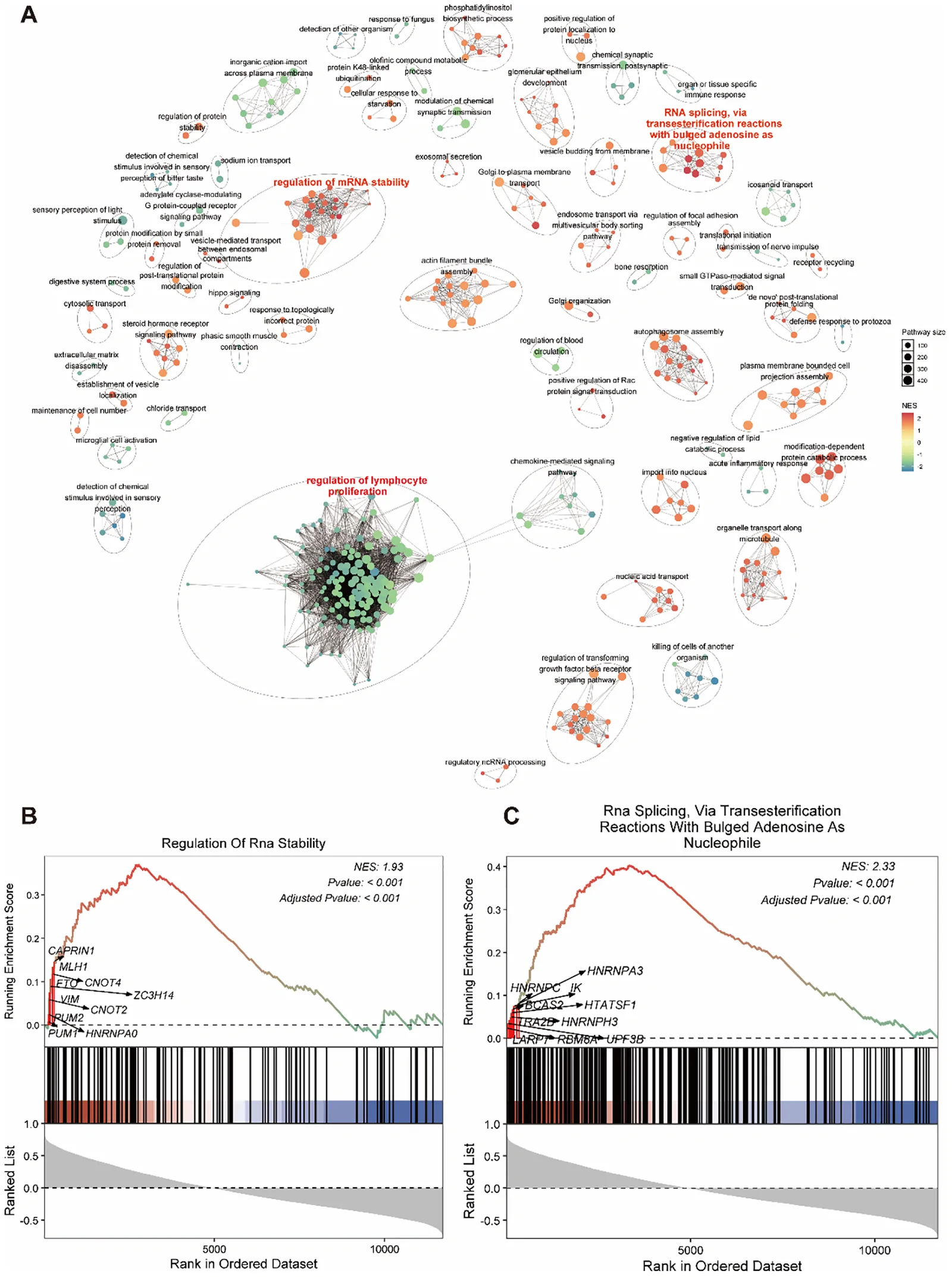
Gene Set Enrichment Analysis (GSEA) reveals SSB-mediated orchestration of RNA processing. **(A)** Global GSEA profile based on the transcriptomic correlation with SSB expression in the GSE30122 cohort. The plot highlights significantly upregulated pathways (e.g., Regulation of RNA Stability, RNA Splicing) and downregulated pathways (e.g., Regulation of Lymphocyte Proliferation). **(B, C)** Specific GSEA mountain plots illustrating the robust positive enrichment of the “Regulation of RNA Stability” **(B)** and “RNA Splicing, Via Transesterification Reactions With Bulged Adenosine As Nucleophile” **(C)** signaling pathways in samples with high SSB expression.

Investigating the broader molecular mechanisms underlying SSB-mediated R-loop regulation prompted us to evaluate its transcriptomic co-expression networks. Using the bulk RNA-seq matrix (GSE30122) (17), we computed the correlation coefficients between *SSB* and all other expressed genes. Ranking the entire transcriptome based on these correlation coefficients, we performed Gene Set Enrichment Analysis (GSEA) to uncover the biological cascades coordinated by SSB. The global GSEA profile revealed that genes positively correlated with *SSB* were robustly enriched in critical RNA processing pathways, specifically the “Regulation of RNA Stability” and “RNA Splicing, Via Transesterification Reactions with Bulged Adenosine as Nucleophile” (**Figure 5A**). The highly significant enrichment of these specific modules was further detailed in individual enrichment plots (**Figures 5B, C**). Conversely, immune-related pathways, such as the “Regulation of Lymphocyte Proliferation,” were significantly downregulated in association with *SSB* expression (**Figure 5A**). Given that defective co-transcriptional RNA splicing and compromised RNA stability are primary catalysts for the pathological hybridization of RNA and DNA, these findings mechanistically suggest that SSB maintains R-loop homeostasis fundamentally by orchestrating proper RNA processing in podocytes.

**Figure 6:**
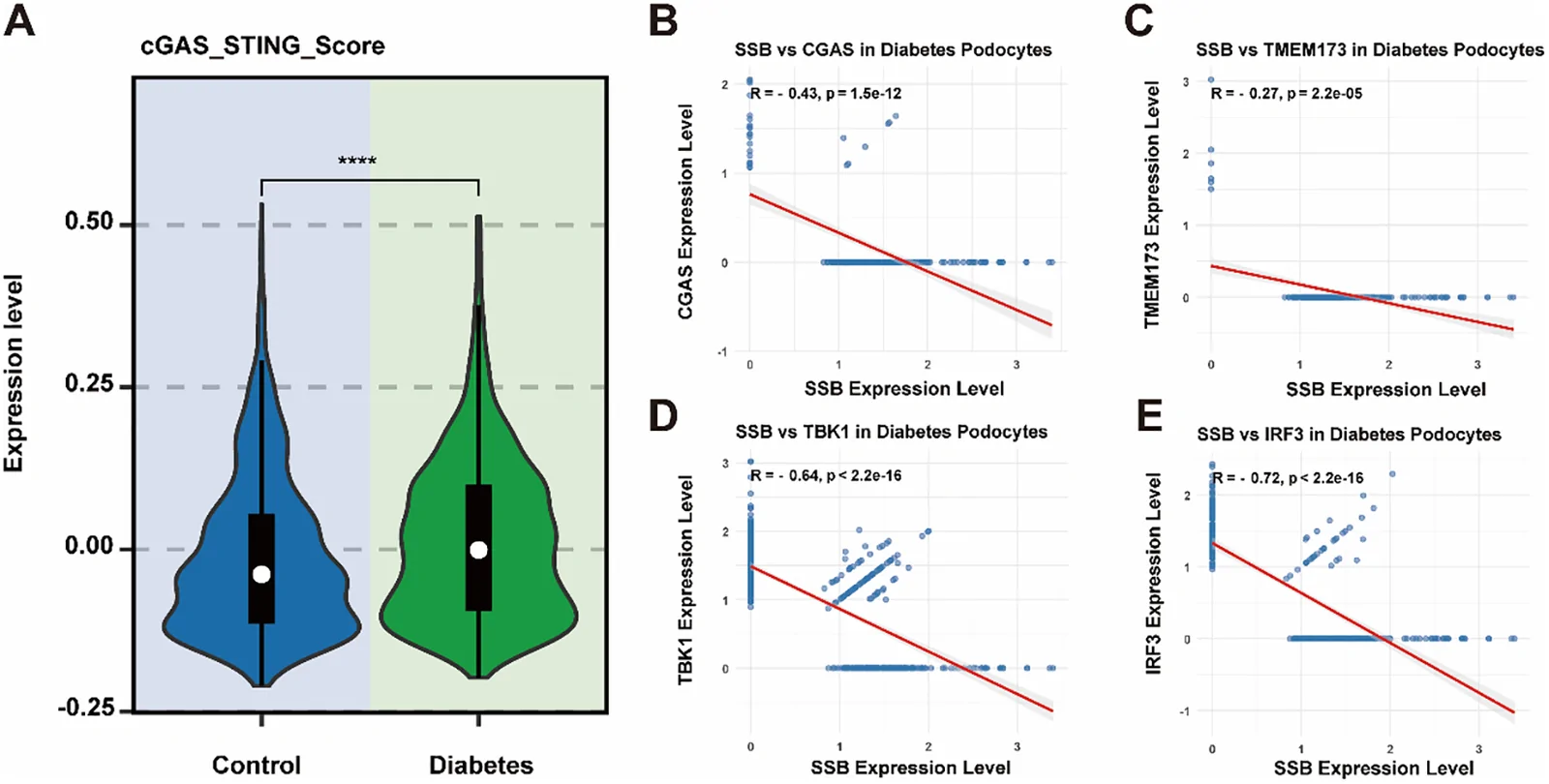
SSB deficiency correlates with the activation of the cGAS-STING inflammatory cascade in diabetic podocytes. **(A)** Violin plot indicating the significant amplification of the cGAS-STING pathway activation score in diabetic podocytes compared to controls. **(B-E)** Single-cell resolution density scatter plots demonstrating a profound negative correlation between SSB expression and the core transducers of the cGAS-STING inflammatory axis, including CGAS **(B)**, TMEM173/STING1 **(C)**, TBK1 **(D)**, and IRF3 **(E)** in the DKD group. P-values and Spearman’s rho (R) are indicated in each panel.

Emerging literature indicates that unresolved R-loops and their associated DNA double-strand breaks can result in the leakage of nucleic acids into the cytosol (11, 12). These misplaced nucleic acids act as danger-associated molecular patterns (DAMPs) that potently activate the cGAS-dependent innate immune sensing pathway. To determine whether this genomic instability translates into intrinsic inflammation in DKD, we evaluated the transcriptional activity of the cGAS-STING signaling cascade within the single-cell landscape. Module scoring demonstrated a striking and highly significant amplification of the cGAS-STING pathway score in diabetic podocytes compared to the control group (**Figure 6A**), indicating a robust activation of the cytosolic DNA-sensing alarm under high-glucose conditions.

Consistent with the premise that SSB acts as a critical R-loop resolver and RNA stabilizer, its depletion should theoretically exacerbate R-loop accumulation and subsequently hyperactivate this inflammatory cascade. Correlation analysis at the single-cell resolution perfectly validated this hypothesis. *SSB* expression exhibited a profound and highly significant *negative* correlation with the core signal transducers of the cGAS-STING axis, namely *CGAS* (R = -0.43), *TMEM173* (STING, R = -0.27), *TBK1* (R = -0.64), and *IRF3* (R = -0.72) (**Figures 6B-E**) is striking inverse relationship cohesively bridges the upstream RNA/R-loop defect with the downstream inflammatory phenotype. Collectively, these computational analyses establish a robust mechanistic rationale for our subsequent experimental validations: the diabetic milieu induces SSB deficiency, which compromises RNA splicing and R-loop resolution, ultimately culminating in the activation of cGAS-dependent inflammation in podocytes.

### SSB is reduced in glomeruli of diabetic mice and in HG-treated podocytes

Based on our previous observation that Sjogren syndrome antigen B (*SSB*) is markedly and selectively downregulated in podocytes in diabetic kidney disease (**Figure 4B**), we reasoned that loss of SSB may be closely associated with podocyte injury and inflammation, and the progression of DKD. We therefore subjected this finding to experimental validation.

**Figure 7:**
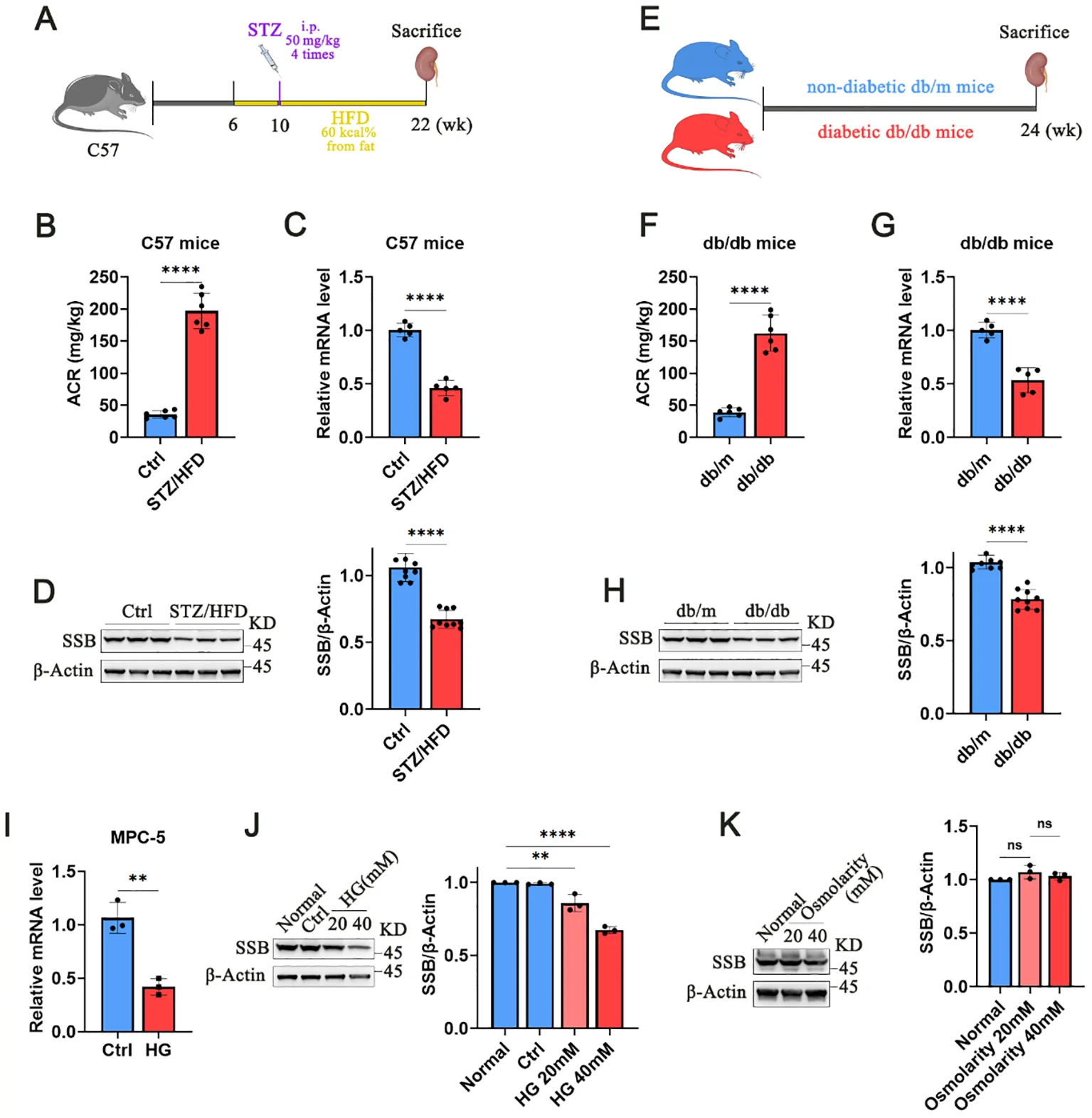
SSB is reduced in glomeruli from two diabetic nephropathy mouse models and in high glucose (HG)-treated podocytes. **(A)** A schematic diagram showing the construction of the STZ/HFD-induced diabetic mice model. **(B)** Urine albumin-to-creatinine ratio (ACR) of mice in WT groups and STZ/HFD‐induced DKD groups (mice *n* = 6). **(C)** Relative mRNA level of *Ssb* in the glomeruli from WT mice and STZ/HFD‐induced DKD mice (mice *n* = 5). **(D)** Western blot analysis and quantification of SSB and β-Actin expression in the glomeruli from WT mice and STZ/HFD‐induced DKD mice (mice *n* = 9). **(E)** A schematic diagram showing the construction of spontaneous type 2 diabetic db/db mice. **(F)** Urine albumin-to-creatinine ratio (ACR) of mice in db/m and db/db groups (mice *n* = 6). **(G)** Relative mRNA level of *Ssb* in the glomerul from db/m and db/db mice (mice *n* = 5). **(H)** Western blot analysis and quantification of SSB and β-Actin expression in the glomeruli from db/m and db/db mice (mice *n* = 9). **(I)** Relative mRNA level of *Ssb* in the MPC-5 cells cultured in control medium (5.5 mM glucose + 34.5 mM mannitol) or 40 mM HG medium (5.5 mM glucose + 34.5 mM glucose) for 48 h (*n* = 3). **(J)** Western blot analysis and quantification of SSB and β-Actin expression in the MPC-5 cells cultured in normal medium (5.5 mM glucose), control medium (5.5 mM glucose + 34.5 mM mannitol), 20 mM HG medium (5.5 mM glucose + 14.5 mM glucose) or 40 mM HG medium (5.5 mM glucose + 34.5 mM glucose) for 48 h (*n* = 3). **(K)** Western blot analysis and quantification of SSB and β-Actin expression in the MPC-5 cells cultured in normal medium (5.5 mM glucose), 20mM Osmolarity (5.5 mM glucose + 14.5 mM mannitol) or 40mM Osmolarity (5.5 mM glucose + 34.5 mM mannitol) for 48 h (*n* = 3). DKD, diabetic kidney disease; HFD, high‐fat diet; HG, high glucose; STZ, streptozotocin. Data are representative of three independent experiments **(D, H, J, K)**. Error bars are shown as mean ± SD with *n* = 3 **(I, J, K)**, *n* = 5 **(C, G)**, *n* = 6 **(B, F)** or *n* = 9 **(D, H)** biological replicates. Two-tailed unpaired Student’s t-test **(B-D, F-I)**. One-way ANOVA with Tukey’s multiple comparisons test **(J, K)**. *P < 0.05, **P < 0.01, ***P < 0.001, ****P < 0.0001, NS, not significant.

We first established a streptozotocin/high-fat diet (STZ/HFD)-induced murine model of DKD (**Figure 7A**). Compared with control animals, STZ/HFD-treated mice exhibited a marked increase in urinary albumin excretion (**Figure 7B**), confirming successful induction of glomerular injury and renal functional impairment. Subsequent analysis of isolated glomeruli revealed that, both *Ssb* mRNA and SSB protein levels were significantly reduced in the glomeruli of STZ/HFD-induced DKD group relative to controls (**Figures 7C, D**). To corroborate the generalizability of this observation, we examined an additional DKD model—the *db/db* mouse, a spontaneous model of type 2 diabetes (**Figure 7E**). At 24 weeks of age, *db/db* mice displayed significantly elevated urinary albumin levels compared with control littermates (**Figure 7F**). Further assessment confirmed a pronounced downregulation of *Ssb* mRNA and SSB protein expression in glomeruli isolated from *db/db* mice (**Figures 7G, H**). Collectively, these results demonstrate that *Ssb*/SSB expression in glomeruli is markedly suppressed during the pathogenesis of DKD.

Moreover, *in vitro* experiments using conditionally immortalized mouse podocytes (MPC-5 cells) revealed that exposure to high glucose (HG) was sufficient to induce a significant reduction in SSB expression (**Figures 7I, J**). To exclude the possibility that this reduction was caused by hyperosmolality, we performed osmotic control experiments using mannitol at concentrations that matched the added osmolarity of the high-glucose conditions (14.5 mM and 34.5 mM mannitol in low-glucose medium, corresponding to the 20 mM and 40 mM glucose groups, respectively). Mannitol treatment did not significantly alter SSB expression compared with the low-glucose control, confirming that the effect is specifically due to high glucose rather than increased osmolarity (**Figure 7K**). Taken together, these findings indicate that podocyte *Ssb*/SSB expression within the glomerulus is substantially downregulated during the development of diabetic kidney disease.

### SSB deficiency drives podocyte inflammation in DKD

**Figure 8:**
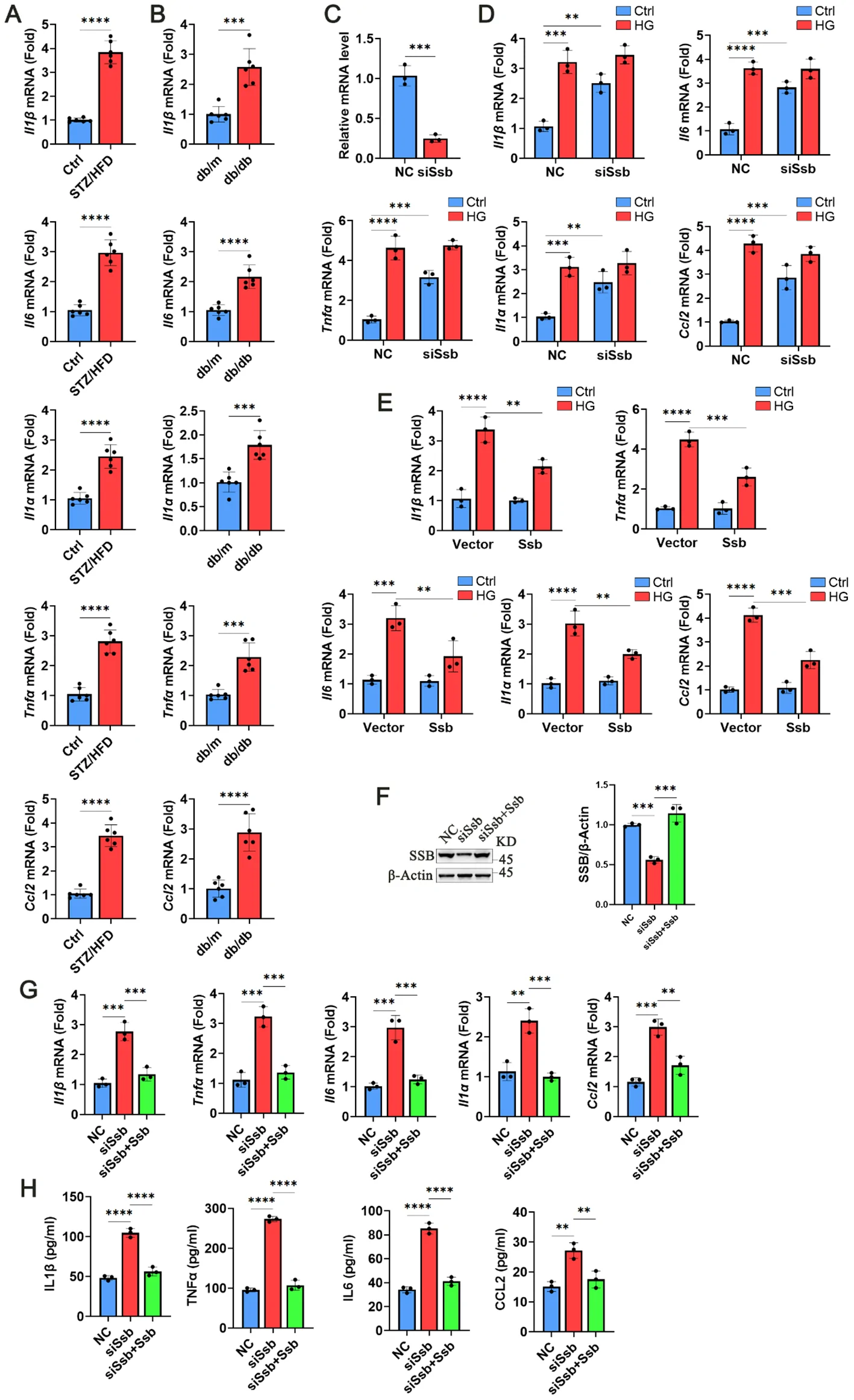
Knockdown of SSB in podocytes exacerbates, whereas overexpression ameliorates, glomerular inflammation under diabetic conditions. **(A)** Relative mRNA level of *Ilβ, Il6, Il1α, Tnfα, Ccl2* in the glomeruli from WT mice and STZ/HFD‐induced DKD mice (mice *n* = 6). **(B)** Relative mRNA level of *Ilβ, Il6, Il1α, Tnfα, Ccl2* in the glomeruli from db/m and db/db mice (mice *n* = 6). **(C)** Relative mRNA level of *Ssb* in the MPC-5 cells after knockdown of SSB with siRNA for 48 h (*n* = 3). **(D)** Relative mRNA level of *Ilβ, Il6, Il1α, Tnfα, Ccl2* in the MPC-5 cells transfected with siRNA against *SSB* for 24 h, followed by culture in control medium (5.5 mM glucose + 34.5 mM mannitol) or 40 mM HG medium (5.5 mM glucose + 34.5 mM glucose) for an additional 48 h (*n* = 3). **(E)** Relative mRNA level of *Ilβ, Il6, Il1α, Tnfα, Ccl2* in the MPC-5 cells overexpressing of *SSB* for 24 h, followed by cultured in control medium (5.5 mM glucose + 34.5 mM mannitol) or 40 mM HG medium (5.5 mM glucose + 34.5 mM glucose) for an additional 48 h (*n* = 3). **(F)** Western blot analysis and quantification of SSB and β-Actin were conducted in control MPC-5 cells, cells subjected to SSB knockdown, and cells in which SSB was overexpressed subsequent to SSB knockdown (*n* = 3). **(G)** Relative mRNA level of *Ilβ, Il6, Il1α, Tnfα, Ccl2* in MPC-5 cells under the indicated treatment conditions, as shown in Panel F (*n* = 3). **(H)** ELISA was used to detect the levels of inflammatory cytokines IL-1β, TNF-α, IL-6 and CCL2 in the supernatant of MPC5 cells under the indicated treatment conditions (*n* = 3). Data are representative of three independent experiments **(F)**. Error bars are shown as mean ± SD with *n* = 3 **(C-H)** or *n* = 6 **(A, B)** biological replicates. Two-tailed unpaired Student’s t-test **(A-C)**. One-way ANOVA with Tukey’s multiple comparisons test **(D-H)**. *P < 0.05, **P < 0.01, ***P < 0.001, ****P < 0.0001, NS, not significant.

Given that podocyte-driven inflammation is thought to play a critical role in the pathogenesis of diabetic kidney disease, and that we previously found a significant negative correlation between SSB expression and key molecules of the inflammation-associated cGAS-dependent inflammatory signaling (**Figures 6B-E****),** we hypothesized that downregulation of SSB is a principal driver of podocyte inflammation during DKD progression. To investigate this relationship, we assessed the expression of a panel of pro-inflammatory mediators—*Il1b*, *Il6*, *Il1a*, *Tnf*, and *Ccl2*—in glomeruli isolated from two distinct murine models of DKD. Consistent with expectations, transcript levels of these inflammatory factors were markedly elevated in glomeruli of diabetic mice relative to non-diabetic controls (**Figures 8A, B**).

We next sought to determine whether the downregulation of SSB expression observed in the context of DKD (**Figure 7**) is functionally linked to the activation of podocyte inflammatory responses. To this end, we first achieved efficient knockdown of *Ssb* in conditionally immortalized mouse podocytes (MPC-5 cells) using siRNA (**Figure 8C**). Subsequent exposure of podocytes to HG conditions resulted in a significant upregulation of the aforementioned inflammatory mediators, recapitulating the findings obtained *in vivo* (**Figure 8D**). Strikingly, siRNA-mediated depletion of *Ssb* alone, even in the absence of HG stimulation, was sufficient to induce a comparable elevation in inflammatory gene expression (**Figure 8D**). These data strongly suggest that SSB deficiency directly promotes podocyte inflammation.

To further substantiate the specific contribution of SSB to HG-induced inflammatory signaling, we performed gain-of-function experiments. Overexpression of SSB via plasmid transfection significantly attenuated the HG-triggered induction of *Il1b*, *Il6*, *Il1a*, *Tnfa*, and *Ccl2* (**Figure 8E**). To definitively establish the causative role of SSB depletion in driving podocyte inflammation, we conducted a rescue experiment. We successfully knocked down endogenous *Ssb* and subsequently restored SSB expression in the knockdown background (**Figure 8F**). Whereas *Ssb* knockdown alone led to a robust increase in the expression of inflammatory cytokines, reconstitution of SSB expression in these cells fully reversed the pro-inflammatory phenotype, restoring cytokine levels toward baseline (**Figure 8G**). Moreover, ELISA analysis of IL-1β, IL-6, TNF-α, and CCL2 in culture supernatants confirmed that Ssb knockdown markedly increased the secretion of all four cytokines compared with the NC group, whereas restoration of SSB expression largely reversed this effect, reducing cytokine levels toward baseline (**Figure 8H**). These secretion data are in full agreement with the mRNA changes and directly demonstrate that SSB knockdown drives the release of inflammatory mediators from podocytes.

Collectively, these findings demonstrate that the induction of podocyte inflammation during the progression of diabetic kidney disease is mechanistically mediated by the downregulation of SSB expression.

### SSB downregulation triggers R-loop accumulation and cGAS-mediated inflammation in podocytes

**Figure 9:**
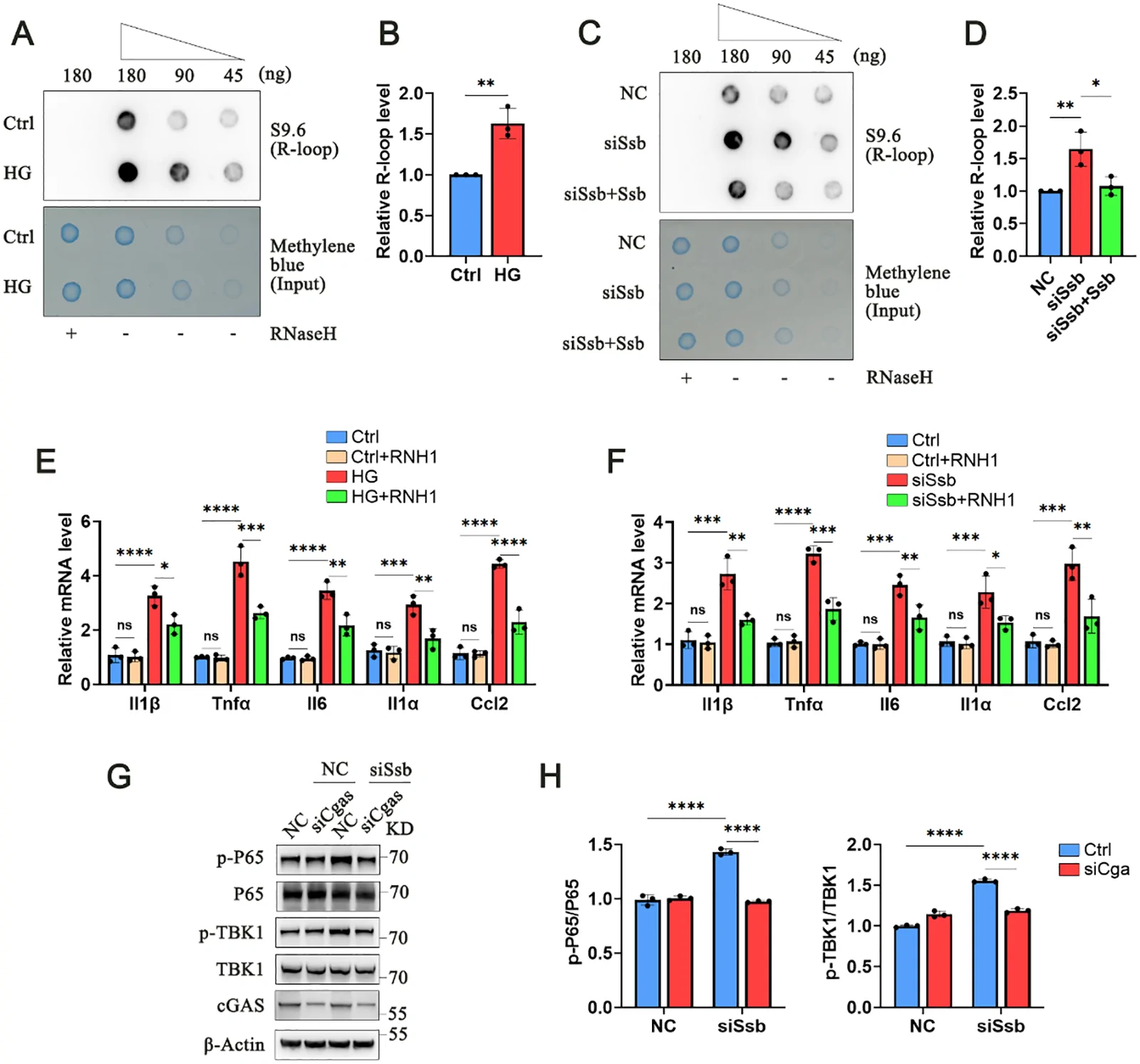
HG-induced SSB loss triggers cGAS inflammation via R-loop accumulation. **(A)** Dot blot analysis of R-loop levels in MPC-5 cells cultured in control medium (5.5 mM glucose + 34.5 mM mannitol) or 40 mM HG medium (5.5 mM glucose + 34.5 mM glucose) for 48 h. DNA from parallel samples was treated with RNase H as indicated, followed by dot blot analysis. Methylene blue staining was used as control. **(B)** Quantification of dot blot signal intensity in **(A)** Values represent the mean of three concentrations for the control or HG treatment (*n* = 3). **(C)** Dot blot analysis of R-loop levels was conducted in control MPC-5 cells, cells subjected to SSB knockdown, and cells in which SSB was overexpressed subsequent to SSB knockdown. DNA from parallel samples was treated with RNase H as indicated, followed by dot blot analysis. Methylene blue staining was used as control. **(D)** Quantification of dot blot signal intensity in **(C)** Values represent the mean of three concentrations for the NC, siSsb or siSsb+Ssb treatment (*n* = 3). **(E)** Relative mRNA levels of *Il1β*, *Il6*, *Il1α*, *Tnfα*, and *Ccl2* were quantified in control MPC-5 cells (5.5 mM glucose + 34.5 mM mannitol), control cells (5.5 mM glucose + 34.5 mM mannitol) overexpressing *Rnaseh1* (RNH1) for 24 h, cells exposed to HG (5.5 mM glucose + 34.5 mM glucose) for 48 h, and cells overexpressing *Rnaseh1* (RNH1) for 24 h prior to an additional 48 h of HG (5.5 mM glucose + 34.5 mM glucose) exposure (*n* = 3). **(F)** Relative mRNA levels of *Il1β*, *Il6*, *Il1α*, *Tnfα*, and *Ccl2* were quantified in control MPC-5 cells, control cells with subsequent *Rnaseh1* (RNH1) overexpression, cells subjected to SSB knockdown, and SSB-knockdown cells with subsequent *Rnaseh1* (RNH1) overexpression (*n* = 3). **(G)** Western blot analysis of p-P65, P65, p-TBK1, TBK1, cGAS and β-Actin was performed in MPC-5 cells transfected with the indicated siRNA combinations: NC + NC, NC + siCgas, siSsb + NC, and siSsb + siCgas. **(H)** Quantification of western blot signal intensity in G (*n* = 3). Data are representative of three independent experiments **(A, C, G)**. Error bars are shown as mean ± SD with *n* = 3 **(B, D, E-F, H)** biological replicates. Two-tailed unpaired Student’s t-test **(B)**. One-way ANOVA with Tukey’s multiple comparisons test **(D, E, F, H)**. *P < 0.05, **P < 0.01, ***P < 0.001, ****P < 0.0001, NS, not significant.

Given our previous observation that SSB exhibited the strongest positive correlation with the R-loop resolver score (**Figures 3L–N**), and that gene set enrichment analysis (GSEA) revealed that SSB predominantly mediates RNA processing and splicing in DKD (**Figures 5A-C**), we hypothesized that SSB may influence DKD progression through these processes. Transcription and splicing are highly coupled processes in eukaryotes, and perturbations to the host splicing machinery frequently disrupt the homeostatic balance of R-loops during transcription (8, 9). Indeed, previous studies have demonstrated that depletion of splicing-associated RNA-binding proteins (RBPs) promotes increased R-loops (9). We therefore examined changes in R-loop levels in podocytes following exposure to high glucose (HG). Notably, HG treatment resulted in a significant accumulation of R-loops in genomic DNA derived from podocytes (**Figures 9A, B**), confirming that R-loop burden is elevated during the pathogenesis of DKD.

To ascertain whether this increase in R-loops is specifically mediated by the reduction in SSB expression observed in DKD, we performed loss- and gain-of-function experiments in podocytes. Consistent with our hypothesis, siRNA-mediated knockdown of *Ssb* led to a marked elevation in R-loop levels (**Figures 9C, D**). Crucially, restoration of SSB expression in the *Ssb*-knockdown background effectively reversed this accumulation, returning R-loop levels toward baseline (**Figures 9C, D**). These data establish that SSB downregulation is both necessary and sufficient to drive R-loop accumulation in the context of DKD.

We next investigated whether the podocyte inflammation induced by SSB deficiency is causally linked to the concomitant increase in R-loops. To address this, we utilized a plasmid encoding murine Rnaseh1 (RNH1), an enzyme that specifically resolves RNA-DNA hybrids within R-loop structures (18). Overexpression of RNH1 in podocytes significantly attenuated HG-induced upregulation of inflammatory mediators (**Figure 9E**). Moreover, RNH1 overexpression similarly abrogated the inflammatory response triggered by *Ssb* knockdown alone (**Figure 9F**). Collectively, these findings demonstrate that SSB downregulation in DKD promotes podocyte inflammation via an R-loop-dependent mechanism.

Emerging evidence indicates that the accumulation of R-loops can activate cGAS-mediated innate immune signaling (11, 12). In diabetic podocytes, the cGAS-dependent inflammatory signaling pathway score was markedly and highly significantly elevated (**Figure 6A**), and low SSB expression showed a significant negative correlation with the activation of the cGAS-dependent inflammatory signaling (**Figures 6B-E**). To determine whether the inflammatory phenotype resulting from SSB loss and R-loop accumulation is transduced through the cGAS-dependent inflammatory signaling pathway, we performed double-knockdown experiments in podocytes. Notably, depletion of *Cgas* significantly blunted the activation of downstream inflammatory signaling cascades induced by *Ssb* knockdown (**Figures 9G, H**).

In summary, our findings delineate a novel pathogenic axis in diabetic kidney disease wherein downregulation of SSB drives the accumulation of R-loops, which in turn triggers cGAS-dependent inflammatory signaling in podocytes, thereby fueling disease progression.

## Discussion

The present study uncovers a previously unrecognized pathogenic axis in diabetic kidney disease, wherein in cultured podocytes the loss of the R-loop resolver SSB triggers aberrant R-loop accumulation, which in turn activates the cGAS-dependent innate immune pathway to drive podocyte inflammation. Our integrative strategy—combining single-cell transcriptomic profiling, machine learning-based biomarker discovery, and targeted experimental validation—provides convergent evidence suggesting that disrupted R-loop homeostasis may be a key contributor to podocytopathy and sterile inflammation in DKD. These findings fundamentally expand the molecular framework of podocyte injury and nominate R-loop surveillance as a potential therapeutic vulnerability.

Diabetic kidney disease has long been conceptualized as a podocyte-centric disorder (4, 5), yet the upstream events that couple cellular stress to inflammatory activation have remained incompletely defined. Our data now position pathological R-loop accumulation as a critical missing link. Podocytes are post-mitotic cells with an exceptionally high transcriptional burden required to sustain the glomerular filtration barrier (19, 20); this transcriptional demand renders them acutely susceptible to perturbations in R-loop dynamics. We demonstrate that under high-glucose conditions, podocytes suffer a dual-directional disruption of R-loop homeostasis—impaired resolution capacity coupled with enhanced formation propensity—which collectively results in net R-loop accumulation. This finding aligns with the emerging recognition that R-loop dysregulation is a hallmark of genomic instability in metabolically stressed cells, yet extends this concept for the first time to the renal glomerulus.

A key contribution of our study is the identification of SSB (Sjögren syndrome antigen B/La protein) as a podocyte-selective factor required for R-loop homeostasis whose deficiency precipitates this pathogenic cascade. SSB has been classically characterized as an RNA-binding protein (RBP) that associates with the 3′ termini of nascent RNA polymerase III transcripts, protecting them from exonucleolytic degradation and facilitating proper RNA processing (21). However, its role in regulating R-loop dynamics has not been previously appreciated. Our multi-level computational analyses robustly position SSB within the R-loop resolution machinery: it exhibited the strongest positive correlation with the resolver module among all candidates, and its expression was selectively and profoundly downregulated in diabetic podocytes, a pattern not observed in other renal cell types. Mechanistically, GSEA revealed that SSB-associated transcriptional programs are dominated by RNA splicing and RNA stability pathways—two processes intimately coupled to R-loop suppression. Defective co-transcriptional splicing and compromised RNA stability are well-established triggers for the formation of stable RNA: DNA hybrids, as the nascent transcript is not properly packaged into ribonucleoprotein particles and instead re-anneals with the template DNA strand (9, 22). Thus, our data suggest a model wherein SSB loss impairs proper RNA biogenesis, leading to the persistence of unprocessed transcripts that pathologically hybridize with the DNA template, forming aberrant R-loops. Notably, RBPs commonly recruit R-loop-resolving enzymes during co-transcriptional splicing to promote R-loop resolution (23, 24). We hypothesize that SSB may similarly facilitate R-loop elimination by recruiting such enzymes during RNA biogenesis. In summary, our data suggest a dual model: SSB deficiency directly causes R-loop formation by destabilizing nascent RNA, and indirectly impedes R-loop clearance by disrupting the recruitment of canonical R-loop resolvases. This mechanistic framework not only elucidates the specific role of SSB in the kidney but also expands the functional repertoire of this protein beyond its canonical activities in RNA metabolism.

Our results further demonstrate that SSB knockdown-induced R-loop accumulation in MPC5 cells *in vitro* triggers the activation of cGAS-dependent innate immune signaling. The cGAS-dependent inflammatory signaling pathway has emerged as a pivotal sensor of cytosolic DNA, and recent studies have established that unresolved R-loops and associated DNA damage can result in the leakage of self-nucleic acids into the cytoplasm, where they serve as potent DAMPs (12). We provide correlative and functional evidence that this axis is operative in podocytes: diabetic podocytes exhibit a robust activation signature of the cGAS-dependent inflammatory signaling, SSB expression inversely correlates with core pathway components, and, most critically, both RNase H1-mediated resolution of R-loops and genetic depletion of Cgas effectively abrogated the inflammatory phenotype triggered by SSB loss. These rescue experiments illustrate a possible inflammatory activation pathway: SSB deficiency-R-loop accumulation-cGAS activation-inflammation. This finding is of particular significance because it suggests a mechanism by which a cell-intrinsic metabolic insult (high glucose) is translated into an immunogenic signal within a single cell type, providing a potential mechanistic rationale for the sterile inflammation that characterizes DKD progression.

Two recent studies provide independent support for linking aberrant nucleic acid metabolism to innate immune activation in DKD. In db/db mice, irbesartan restored the expression of circadian rhythm and cell cycle genes (25); since circadian disruption and unscheduled cell cycle re-entry can provoke replication stress and cytosolic nucleic acid accumulation, this renoprotective effect may partly reflect the attenuation of nucleic acid–driven innate immune signaling, consistent with the R-loop–cGAS axis we describe. Single-cell profiling further revealed that dapagliflozin reprograms macrophages toward an anti-inflammatory phenotype and highlighted tubular-immune crosstalk in DKD (26). Our finding that SSB loss–induced R-loop accumulation triggers podocyte-intrinsic cGAS activation suggests that such podocyte-derived inflammatory signals could influence the renal immune landscape, connecting nuclear nucleic acid stress to the downstream immune microenvironment. Together, these studies reinforce a model in which dysregulated nucleic acid metabolism—whether through circadian/cell cycle disturbance or R-loop accumulation—converges on innate immune activation in the diabetic kidney.

The translational implications of this work are multifaceted. First, SSB emerges as a highly sensitive and specific biomarker for diabetic podocytopathy, as evidenced by its exceptional AUC of 0.983 in the training cohort and its selective downregulation in podocytes. We note, however, that this biomarker performance was evaluated in a single training cohort and has not yet been independently validated in external patient populations or in clinically accessible biospecimens such as urine or plasma; therefore, SSB should currently be regarded as a candidate diagnostic biomarker. The cell-type exclusivity of its expression change underscores its utility in liquid biopsy or histological assessments targeting glomerular injury (20, 27). Second, the identified SSB–R-loop–cGAS-dependent inflammatory signaling axis offers multiple potentially druggable nodes. Pharmacological stabilization of SSB, enhancement of R-loop resolution capacity (e.g., via RNase H1 overexpression or small-molecule activators), or targeted inhibition of cGAS-dependent inflammatory signaling could each theoretically interrupt the inflammatory cascade at distinct levels. These therapeutic strategies, while mechanistically plausible, remain speculative at this stage and will require rigorous preclinical testing in podocyte-specific Ssb knockout models and DKD animal models before any clinical translation can be envisioned. Given that podocyte loss is irreversible and current therapies largely focus on hemodynamic and metabolic control, strategies aimed at preserving podocyte genomic integrity and quelling sterile inflammation could represent a paradigm shift. Crucially, unlike conventional anti-inflammatory approaches that carry the risk of systemic immunosuppression, intercepting the R-loop–cGAS-dependent inflammatory signaling axis downstream of SSB loss offers the prospect of precisely extinguishing inflammation at its podocyte-intrinsic source, without globally compromising adaptive immunity. This therapeutic differentiation is reinforced by the fact that small-molecule inhibitors of cGAS are already in clinical development for other inflammatory conditions, which could facilitate drug repurposing for DKD (28).

Several limitations should be acknowledged. First, the upstream molecular mechanism responsible for SSB downregulation in diabetic podocytes has not been delineated; whether the loss occurs through transcriptional silencing, post-transcriptional regulation by microRNAs, or enhanced proteasomal degradation remains an important open question. Additionally, while the high evolutionary conservation of the R-loop regulatory machinery is consistent with potential interspecies translatability, the causal relationships inferred from our data should be interpreted with caution. Definitive *in vivo* causality awaits podocyte-specific *Ssb* knockout or rescue models, which will also be essential to assess the therapeutic potential of targeting this axis and to exclude off-target effects. Furthermore, although rescue experiments overexpressing RNase H1 and depleting cGAS demonstrate that R-loop accumulation and cGAS activation are necessary for SSB loss-induced inflammation in cultured podocytes, our data cannot exclude contributions from other high-glucose stress pathways, such as DNA damage, mitochondrial dysfunction, or nonspecific innate immune activation. Moreover, we acknowledge that our study does not resolve the full cGAS–STING cascade. Although we detected cGAS-dependent TBK1 and NF-κB phosphorylation, key upstream and downstream events — including direct cytosolic DNA sensing, cGAMP production, STING activation, and IRF3-dependent responses such as p-IRF3 or IFNB1 induction — remain to be directly measured in future work. Finally, the precise molecular mechanism by which SSB prevents R-loop accumulation—whether through regulating RNA metabolism during co-transcription, or indirect effects via recruiting R-loop resolved enzymes—remains to be elucidated through biochemical and genome-wide mapping approaches such as DRIP-seq, CLIP-seq, Co-IP.

In summary, our study provides evidence for a novel genomic-stability-driven inflammatory mechanism in diabetic podocytes, wherein SSB depletion disrupts R-loop resolution, leading to activation of the cGAS-dependent inflammatory signaling pathway. These findings suggest that R-loop dysregulation may contribute to DKD beyond its established metabolic and hemodynamic dimensions, and highlight the preservation of R-loop homeostasis as a potential therapeutic strategy. Future *in vivo* intervention studies are needed to substantiate this therapeutic concept and to evaluate its translational viability.

## Methods

### Data acquisition

Single-cell RNA-seq data (GSE131882) and training transcriptomic data (GSE30122) of human glomerular samples were downloaded from GEO. An independent validation dataset (GSE30528) was used to externally validate the diagnostic performance of identified hub genes. The scRNA-seq dataset was utilized for cellular landscape profiling, while the bulk RNA-seq dataset was employed as the training cohort for machine learning and bulk transcriptomic validation. A comprehensive summary of datasets, sample composition, and methodological parameters is provided in (**Supplementary Table 2**).

### Array data processing

After filtering for glomeruli-specific samples, 26 control and 9 diabetic nephropathy samples were retained from GSE30122. Raw CEL files were processed using RMA normalization (Robust Multi-array Average) with the affy package. Probes were annotated using GPL571 platform, and multiple probes mapping to the same gene were collapsed by selecting the probe with highest mean expression.

### Single-cell RNA-seq data processing and cell annotation

Single-cell RNA sequencing data from GSE131882 (6 samples: 3 control, 3 diabetic) was analyzed using Seurat package (v4.3.0). Quality control was performed with thresholds: min.cells=3, min.features=300. Harmony was used for batch correction and integration. Cells with excessive mitochondrial gene expression (>20%) or abnormal feature counts were filtered out to ensure quality. Cell types were annotated using canonical marker genes, with podocytes identified by expression of NPHS1, NPHS2, WT1, and SYNPO (**Supplementary Figure 1**). To eliminate batch effects across different biological samples (*orig.ident*), the Harmony algorithm was applied to integrate the datasets. Uniform Manifold Approximation and Projection (UMAP) was employed for non-linear dimensionality reduction. The clustree package was utilized to determine the optimal clustering resolution (set at 0.5), resulting in 15 initial clusters. Differentially expressed genes (DEGs) for each cluster were computed using the COSG algorithm. Cell type annotation was subsequently performed using the ACT (http://xteam.xbio.top/ACT/) automated annotation tool, supplemented by canonical marker matching, which consolidated the cells into 12 distinct renal cell lineages.

### R-loop regulator classification

R-loop regulatory genes were obtained from R-loopDB database (https://rloopbase.nju.edu.cn/), a curated repository of experimentally validated R-loop factors. Genes were classified into four categories based on their documented functional roles: 1. Resolver/Suppressor: Genes that prevent, limit, or remove R-loops (e.g., RNASEH1, SETX, WRN, BLM). Keywords used: ‘prevent R-loop’, ‘suppress R-loop’, ‘resolve R-loop’, ‘remove R-loop’, ‘degrade RNA: DNA’. 2. Promoter/Facilitator: Genes that promote or stabilize R-loop formation (e.g., ADAR, RPA1, RPA2). Keywords used: ‘promote R-loop’, ‘stabilize R-loop’, ‘facilitate R-loop’.3. Processor/Damage-associated: Genes involved in R-loop processing or associated DNA damage response.4. Ambiguous: Genes with unclear or context-dependent functions; excluded from module scoring. The complete gene list with functional annotations is provided in (**Supplementary Table 3**).

### R-loop module scoring and functional decoupling

To quantify the R-loop regulatory capacity at the single-cell resolution, the AddModuleScore function in Seurat was applied to the podocyte subpopulation using the 1,284 R-loop regulatory genes. To dissect the specific topological mechanisms, we focused on a subset of regulators whose functions had been previously characterized and annotated in the R-loop database, and further classified them based on their documented biological roles. These functionally validated genes were decoupled into two functionally opposing modules: “Rloop_Promoters” (factors facilitating RNA: DNA hybrid formation) and “Rloop_Resolvers” (factors mediating clearance). Module scores for Promoters and Resolvers were calculated separately to evaluate the R-loop homeostatic balance in podocytes. The complete gene lists used for module scoring are provided in **Supplementary Table 4**.

### Machine learning-based feature selection

To identify core diagnostic biomarkers from the intersecting genes (scRNA-seq podocyte DEGs vs. R-loop regulators), dual machine learning algorithms were deployed using the GSE30122 bulk RNA-seq matrix. First, the Least Absolute Shrinkage and Selection Operator (LASSO) regression with 10-fold cross-validation. Lambda.min was selected as the optimal penalty parameter based on minimum cross-validation error. Random Forest analysis (randomForest package, v4.7-1.1) with 500 trees was subsequently applied to rank the importance of selected genes using MeanDecreaseGini metric. The diagnostic performance was evaluated using ROC curves with 95% confidence intervals calculated via bootstrap method (2000 iterations). The intersection of genes selected by both LASSO and RF defined the ultimate hub genes.

### Gene set enrichment analysis

To elucidate the biological pathways orchestrated by the hub gene *SSB*, a single-gene GSEA was conducted. Pearson correlation coefficients between *SSB* and all other expressed genes in the GSE30122 dataset were calculated. The entire transcriptome, ranked in descending order by correlation coefficient, was subjected to GSEA using the clusterProfiler R package. Pathways from the MSigDB hallmark and biological process (GO-BP) collections were evaluated, and statistical significance was defined as an adjusted p-value < 0.05.

### STZ/HFD-induced diabetic mice

All mice were maintained under controlled environmental conditions, including a 12‐h light/dark cycle and constant temperature of 25 ± 1 °C, with free access to autoclaved water and standard laboratory chow throughout the study period. Six-week-old male mice were fed an HFD (60 kcal% from fat, Research Diets, #D12492) or a control diet (Research Diets, #D12450J) for 4 weeks. Mice were fasted for 4 h and then intraperitoneally injected with streptozotocin (STZ) (50 mg/kg body weight daily for 4 days; #S0130, Sigma, USA) to induce partial insulin deficiency. All the mice were allowed unrestricted access to water and food, followed by continued HFD feeding for an additional 12 weeks. After collecting 24 h urine samples, the mice were euthanized, and the kidney samples were harvested for biochemical analyses. Animal experiments were conducted in accordance with the protocols approved by the Animal Ethics Committee of the 960th Hospital of PLA (approval no. P2024-090).

### Spontaneous type 2 diabetic db/db mice

The male diabetic db/db mice with C57BLKS/JGpt background and matched non‐diabetic db/m littermates were obtained from GemPharmatech (Nanjing, China). They were maintained on a standard chow diet until they reached the ages of 24 weeks. After collecting 24 h urine sample, the mice were euthanized, and the kidney samples were harvested for biochemical analyses. Animal experiments were conducted in accordance with the protocols approved by the Animal Ethics Committee of the 960th Hospital of PLA (approval no. P2024-090).

### Isolation of glomeruli

Glomeruli were harvested using an adapted sieving protocol as previously described (29, 30). Prior to kidney excision, the organ was perfused with sterile Hanks’ balanced salt solution (HBSS). The renal tissue was minced into approximately 1mm^3^ fragments with a scalpel and then subjected to enzymatic digestion at 37 °C for 30 minutes in a solution consisting of collagenase II (Roche, #10103586001), proteinase E (Sigma, #P6911), and deoxyribonuclease I (Sigma, #D4527). Following digestion, the homogenate was sequentially passed through 100-µm (BD, USA) and 70-µm (BD, USA) cell strainers, and finally through a 40-µm strainer (Greiner Bio-one, Germany). Glomeruli were retained on the upper surface of the 40-µm mesh. The resulting suspension was centrifuged at 200 × g for 5 minutes at 4 °C, after which the pelleted material was resuspended in 5 mL of culture medium.

### Cell culture and treatment

The conditionally immortalized mouse podocyte cell line (MPC-5), obtained from Procell (Wuhan, China; Cat. No. CL-0855), was cultured in the high-glucose Dulbecco’s modified Eagle’s medium (Gibco, USA) supplemented with 10% fetal bovine serum (Gibco, USA), 100 U/mL of penicillin and 100 μg/mL streptomycin. All cells were cultured at 37 °C and 5% CO2. For high-glucose (HG) treatment, after seeding, the culture medium was replaced with low-glucose DMEM (5.5 mM glucose; Gibco, USA), which served as the normoglycemic control. High-glucose conditions were then established by supplementing this low-glucose medium with additional glucose to final concentrations of 20 mM (i.e., addition of 14.5 mM glucose) or 40 mM (addition of 34.5 mM glucose). To control for the associated increase in osmolarity, mannitol was added to low-glucose DMEM at equimolar concentrations: 14.5 mM mannitol for the 20 mM glucose group, and 34.5 mM mannitol for the 40 mM glucose group.

### Cell transfection and interference

The coding sequences of Ssb (mRNA ID: NM_001110145) and Rnaseh1 (mRNA ID: NM_011275) were amplified from MPC-5 complementary DNAs. Then, the CDS of *Ssb* and *Rnaseh1* was cloned into a pcDNA 3.1 vector. Plasmids were transiently transfected into MPC-5 cells with Lipofectamine 3000 Reagent (ThermoFisher) according to the manufacturer’s instructions.

To silence the *Ssb* and *Cgas* genes, MPC-5 cells were transfected with mouse *Ssb* siRNA (si*La/Ssb*; Cat. No. sc-40916; Santa Cruz) and mouse *Cgas* siRNA (si*Cgas*; Cat. No. sc-143253; Santa Cruz) respectively. For control cells, MPC-5 cells were transfected with control siRNA (control siRNA; Cat. No. sc-37007; Santa Cruz). Cells were plated in plates at 50−60% confluency and transfected with RNAiMAX (Thermo Fisher, Invitrogen) transfection reagent and 60 nM siRNA.

### RNA extraction and quantitative real-time RT-PCR

Total RNA was extracted from the glomeruli or MPC-5 cells using TRIzol reagent (Thermo Fisher, Invitrogen, 15596026CN), and the RNA was reverse transcribed to synthesize cDNA using HiScript III All-in-one RT SuperMix Perfect for qPCR (Vazyme, R333-01) following the manufacturer’s instructions. Quantitative real-time PCR (qRT-PCR) was conducted using ChamQ Universal SYBR qPCR Master Mix (Vazyme, Q711) on a LightCycler-480 Instrument (Roche).

The following qRT-PCR primers were employed:

*β-actin*-F: ACCGTGAAAAGATGACCCAG,
*β-actin*-R: GTACGACCAGAGGCATACAG;
*Il-6*-F: CAGAGGATACCACTCCCAAC,
*Il-6*-R: CAATCAGAATTGCCATTGCC;
*Tnf-α*-F: GTTGTACCTTGTCTACTCCCAG,
*Tnf-α*-R: GGTTGACTTTCTCCTGGTATGAG;
*Il1a*-F: CGAAGACTACAGTTCTGCCATT,
*Il1a*-R: GACGTTTCAGAGGTTCTCAGAG;
*Il1b*-F: GCAACTGTTCCTGAACTCAACT,
*Il1b*-R: ATCTTTTGGGGTCCGTCAACT;
*Ccl2*-F: ATTCTGTGACCATCCCCTCAT,
*Ccl2*-R: TGTATGTGCCTCTGAACCCAC;

Mouse *Ssb* qPCR Primer Pair was purchased from Beyotime (QM21810S). The relative mRNA quantities are expressed as 2^ − ΔΔCT.

### Immunoblot analysis

Total proteins were isolated from the glomeruli or MPC-5 cells with RIPA buffer (Beyotime). Total protein was quantified using the BCA protein assay kit (Thermo Scientific). Proteins were electrophoresed by SDS‐PAGE, transferred to PVDF membranes, blocked with 5% skim milk (60 min, room temperature) and incubated with primary antibodies overnight at 4 °C: anti‐SSB (1:1000; santa cruz, sc-80656), anti‐β-Actin (1:1000; CST, #8457), anti‐TBK1 (1:1000, CST, #3504), anti‐p‐TBK1 (1:1000; Proteintech, Cat No. 82382-2-RR), anti‐p65 (1:1000; absin, CST, #8242), anti‐p‐p65 (1:1000; CST, #3033), anti-cGAS (1:1000; Proteintech, Cat No. 29958-1-AP). After being washed with PBST, the PVDF membranes were incubated with anti- rabbit/mouse IgG, HRP-linked Antibody (1:2000; CST, #7074/#7076) at room temperature for 1 h and then detected with ECL (Thermo Scientific).

### Cytokine measurement

ELISA kits for mouse IL1β (Catalog #MLB00C), TNF (Catalog #MTA00B), CCL2 (Catalog #MJE00B) and IL-6 (Catalog #M6000B) were from R&D Systems. Cytokine concentrations in cell culture supernatants or serum were determined as recommended by the manufacturer.

### Dot blot

Genomic DNA was isolated using phenol–chloroform extraction followed by ethanol precipitation. The purified DNA was then treated with 10 U/ml RNase T1 (Thermo Fisher) and 20 U/ml RNase III (Beyotime) at 37 °C for 1 hour. Then this DNA was incubated with or without 50 U/ml RNase H (NEB) for 2 h at 37 °C. After RNase digestion, the DNA samples were serially diluted and spotted onto a nylon membrane (Beyotime) at the specified amounts. The membrane was subjected to two rounds of UV cross-linking (2000 μJ × 100). For blocking, the membrane was incubated with 5% non-fat dry milk dissolved in TBST for 1 hour at room temperature. It was then probed with mouse anti-DNA–RNA hybrid S9.6 antibody (Kerafast; 1:1000 dilution) in TBST containing 5% bovine serum albumin (BSA) overnight at 4 °C. Following three washes with TBST, the membrane was incubated with horseradish peroxidase (HRP)-conjugated secondary antibody for 1 hour at room temperature. Chemiluminescent signals were developed using an enhanced chemiluminescence (ECL) detection reagent. To assess total DNA loading, the same membrane was stained with 0.2% (w/v) methylene blue. Band intensities were quantified using ImageJ software.

### Statistical analysis

All experiments were conducted in at least three independent biological replicates, and results are presented as the mean ± standard deviation (SD). Technical replicates were used only to ensure measurement consistency and were averaged prior to statistical analysis. For statistical evaluation, a normal distribution of the data was assumed, and analyses were carried out using GraphPad Prism version 8.4 (GraphPad Software, USA). Comparisons between two groups were performed using an unpaired, two-tailed Student’s t-test. For experiments involving more than two groups with a single independent variable, one-way analysis of variance (ANOVA) followed by Tukey’s *post hoc* test was applied. A p value of less than 0.05 was regarded as statistically significant.

## Data Availability

The original contributions presented in the study are included in the article/**Supplementary Material**. Further inquiries can be directed to the corresponding author.

## Supplementary material

Supplementary Figure 1 Expression of podocyte marker genes in single-cell RNA-seq data. Violin plots show expression levels of NPHS1, NPHS2, WT1, and SYNPO across annotated cell clusters. The x-axis lists cell types; the y-axis represents expression values. Violin width indicates cell density at each expression level; white dots and thick black bars denote median and interquartile range, respectively.

## References

[1] Myakala K Wang XX Shults NV Krawczyk E Jones BA Yang X . NAD metabolism modulates inflammation and mitochondria function in diabetic kidney disease. J Biol Chem. (2023) 299:104975. doi: 10.1016/j.jbc.2023.104975 37429506 PMC10413283

[2] Rayego-Mateos S Rodrigues-Diez RR Fernandez-Fernandez B Mora-Fernández C Marchant V Donate-Correa J . Targeting inflammation to treat diabetic kidney disease: The road to 2030. Kidney Int. (2023) 103:282–96. doi: 10.1016/j.kint.2022.10.030 36470394

[3] Zhu Z Cao Y Jian Y Hu H Yang Q Hao Y . CerS6 links ceramide metabolism to innate immune responses in diabetic kidney disease. Nat Commun. (2025) 16:1528. doi: 10.1038/s41467-025-56891-x 39934147 PMC11814332

[4] Martinez Leon V Hilburg R Susztak K . Mechanisms of diabetic kidney disease and established and emerging treatments. Nat Rev Endocrinol. (2026) 22:21–35. doi: 10.1038/s41574-025-01171-3 40935879 PMC13714763

[5] Mohandes S Doke T Hu H Mukhi D Dhillon P Susztak K . Molecular pathways that drive diabetic kidney disease. J Clin Invest. (2023) 133(4):e165654. doi: 10.1172/jci165654 36787250 PMC9927939

[6] Chen J Zhang Q Guo J Gu D Liu J Luo P . Single-cell transcriptomics reveals the ameliorative effect of rosmarinic acid on diabetic nephropathy-induced kidney injury by modulating oxidative stress and inflammation. Acta Pharm Sin B. (2024) 14:1661–76. doi: 10.1016/j.apsb.2024.01.003 38572101 PMC10985035

[7] Li Z Tan D Lin J Zhang T Liu B Zhao B . Podocyte TLR4 deletion alleviates diabetic kidney disease through prohibiting PKCδ/SHP-1-dependent ER stress and relieving podocyte damage and inflammation. J Adv Res. (2026) 82:845–62. doi: 10.1016/j.jare.2025.07.013 40659086 PMC13001067

[8] García-Muse T Aguilera A . R loops: From physiological to pathological roles. Cell. (2019) 179:604–18. doi: 10.1016/j.cell.2019.08.055 31607512

[9] Petermann E Lan L Zou L . Sources, resolution and physiological relevance of R-loops and RNA-DNA hybrids. Nat Rev Mol Cell Biol. (2022) 23:521–40. doi: 10.1038/s41580-022-00474-x 35459910

[10] Brickner JR Garzon JL Cimprich KA . Walking a tightrope: The complex balancing act of R-loops in genome stability. Mol Cell. (2022) 82:2267–97. doi: 10.1016/j.molcel.2022.04.014 35508167 PMC9233011

[11] Bao Q Huang Y Deng M Zhang C Zu D He H . PVC nanoplastics exposure exacerbates asthma through R-loop accumulation and subsequent STING activation in macrophages. Adv Sci (Weinheim Baden-Wurttemberg Germany). (2025) 12:e02223. doi: 10.1002/advs.202502223 40919700 PMC12667527

[12] Crossley MP Song C Bocek MJ Choi JH Kousouros JN Sathirachinda A . R-loop-derived cytoplasmic RNA-DNA hybrids activate an immune response. Nature. (2023) 613:187–94. doi: 10.1038/s41586-022-05545-9 36544021 PMC9949885

[13] Wilson PC Wu H Kirita Y Uchimura K Ledru N Rennke HG . The single-cell transcriptomic landscape of early human diabetic nephropathy. PNAS. (2019) 116:19619–25. doi: 10.1073/pnas.1908706116 31506348 PMC6765272

[14] Mathieson PW . The podocyte as a target for therapies--new and old. Nat Rev Nephrol. (2011) 8:52–6. doi: 10.1038/nrneph.2011.171 22045242

[15] Braun F Mandel AM Blomberg L Wong MN Chatzinikolaou G Meyer DH . Loss of genome maintenance is linked to mTOR complex 1 signaling and accelerates podocyte damage. JCI Insight. (2025) 10(12):e172370. doi: 10.1172/jci.insight.172370 40392611 PMC12220965

[16] Lin R Zhong X Zhou Y Geng H Hu Q Huang Z . R-loopBase: A knowledgebase for genome-wide R-loop formation and regulation. Nucleic Acids Res. (2022) 50:D303–15. doi: 10.1093/nar/gkab1103 34792163 PMC8728142

[17] Woroniecka KI Park AS Mohtat D Thomas DB Pullman JM Susztak K . Transcriptome analysis of human diabetic kidney disease. Diabetes. (2011) 60:2354–69. doi: 10.2337/db10-1181 21752957 PMC3161334

[18] Cerritelli SM Crouch RJ . Ribonuclease H: The enzymes in eukaryotes. FEBS J. (2009) 276:1494–505. doi: 10.1111/j.1742-4658.2009.06908.x 19228196 PMC2746905

[19] Cunanan J Zhang D Peired AJ Barua M . Podocytes in health and glomerular disease. Front Cell Dev Biol. (2025) 13:1564847. doi: 10.3389/fcell.2025.1564847 40342933 PMC12058676

[20] Meliambro K He JC Campbell KN . Podocyte-targeted therapies - progress and future directions. Nat Rev Nephrol. (2024) 20:643–58. doi: 10.1038/s41581-024-00843-z 38724717

[21] Marrella SA Brown KA Mansouri-Noori F Porat J Wilson DJ Bayfield MA . An interdomain bridge influences RNA binding of the human La protein. J Biol Chem. (2019) 294:1529–40. doi: 10.1074/jbc.ra118.003995 30530494 PMC6364776

[22] Chakraborty P Huang JTJ Hiom K . DHX9 helicase promotes R-loop formation in cells with impaired RNA splicing. Nat Commun. (2018) 9:4346. doi: 10.1038/s41467-018-06677-1 30341290 PMC6195550

[23] Nguyen HD Yadav T Giri S Saez B Graubert TA Zou L . Functions of replication protein A as a sensor of R loops and a regulator of RNaseH1. Mol Cell. (2017) 65:832–47. doi: 10.1016/j.molcel.2017.01.029 28257700 PMC5507214

[24] Yuan W Al-Hadid Q Wang Z Shen L Cho H Wu X . TDRD3 promotes DHX9 chromatin recruitment and R-loop resolution. Nucleic Acids Res. (2021) 49:8573–91. doi: 10.1093/nar/gkab642 34329467 PMC8421139

[25] Zhao H Li Z Yan M Ma L Dong X Li X . Irbesartan ameliorates diabetic kidney injury in db/db mice by restoring circadian rhythm and cell cycle. J Trans Internal Med. (2024) 12:157–69. doi: 10.2478/jtim-2022-0049 38799791 PMC11117442

[26] Bai Y Chi K Zhao D Shen W Liu R Hao J . Identification of functional heterogeneity of immune cells and tubular-immune cellular interplay action in diabetic kidney disease. J Trans Internal Med. (2024) 12:395–405. doi: 10.2478/jtim-2023-0130 39360161 PMC11444470

[27] Sanchez-Niño MD Sanz AB Ramos AM Ruiz-Ortega M Ortiz A . Translational science in chronic kidney disease. Clin Sci (London Engl 1979). (2017) 131:1617–29. doi: 10.1042/cs20160395 28667063

[28] Decout A Katz JD Venkatraman S Ablasser A . The cGAS-STING pathway as a therapeutic target in inflammatory diseases. Nat Rev Immunol. (2021) 21:548–69. doi: 10.1038/s41577-021-00524-z 33833439 PMC8029610

[29] Chen Z Zhu Z Liang W Luo Z Hu J Feng J . Reduction of anaerobic glycolysis contributes to angiotensin II-induced podocyte injury with foot process effacement. Kidney Int. (2023) 103:735–48. doi: 10.1016/j.kint.2023.01.007 36731609

[30] Liang W Yamahara K Hernando-Erhard C Lagies S Wanner N Liang H . A reciprocal regulation of spermidine and autophagy in podocytes maintains the filtration barrier. Kidney Int. (2020) 98:1434–48. doi: 10.1016/j.kint.2020.06.016 32603735

