# Supplementary material for: SSB deficiency-induced R-loop accumulation triggers podocyte inflammation in DKD
Source: Front Immunol. 2026 Aug 20;17:1911700. doi: 10.3389/fimmu.2026.1911700 (PMC13537955; doi:10.3389/fimmu.2026.1911700)
Supplement: Supplementary file 1 [file Image1.pdf]

## Supplementary Figures and Figures legends

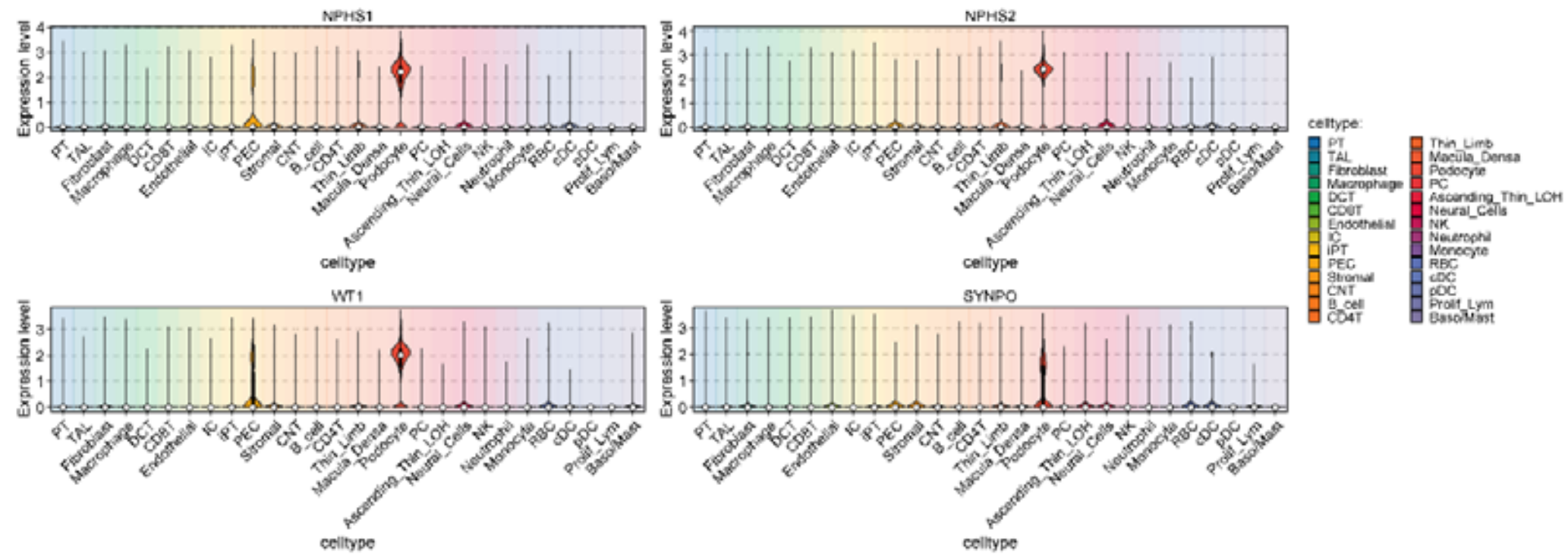


**Supplementary Figure S1. Expression of podocyte marker genes in single-cell RNA-seq data.**

Violin plots show expression levels of NPHS1, NPHS2, WT1, and SYNPO across annotated cell clusters. The x-axis lists cell types; the y-axis represents expression values. Violin width indicates cell density at each expression level; white dots and thick black bars denote median and interquartile range, respectively.
